# Quantifying antimicrobial resistance in food-producing animals in North America

**DOI:** 10.3389/fmicb.2025.1542472

**Published:** 2025-05-27

**Authors:** Mohamed Mediouni, Abdoulaye Baniré Diallo, Vladimir Makarenkov

**Affiliations:** Département d'informatique, Université du Québec á Montréal, Montreal, QC, Canada

**Keywords:** antibiotics, antimicrobial use, antimicrobial resistance, farm animals, food-producing animals, North America

## Abstract

The global misuse of antimicrobial medication has further exacerbated the problem of antimicrobial resistance (AMR), enriching the pool of genetic mechanisms previously adopted by bacteria to evade antimicrobial drugs. AMR can be either intrinsic or acquired. It can be acquired either by selective genetic modification or by horizontal gene transfer that allows microorganisms to incorporate novel genes from other organisms or environments into their genomes. To avoid an eventual antimicrobial mistreatment, the use of antimicrobials in farm animal has been recently reconsidered in many countries. We present a systematic review of the literature discussing the cases of AMR and the related restrictions applied in North American countries (including Canada, Mexico, and the USA). The Google Scholar, PubMed, Embase, Web of Science, and Cochrane databases were searched to find plausible information on antimicrobial use and resistance in food-producing animals, covering the time period from 2015 to 2024. A total of 580 articles addressing the issue of antibiotic resistance in food-producing animals in North America met our inclusion criteria. Different AMR rates, depending on the bacterium being observed, the antibiotic class being used, and the farm animal being considered, have been identified. We determined that the highest average AMR rates have been observed for pigs (60.63% on average), the medium for cattle (48.94% on average), and the lowest for poultry (28.43% on average). We also found that Cephalosporines, Penicillins, and Tetracyclines are the antibiotic classes with the highest average AMR rates (65.86%, 61.32%, and 58.82%, respectively), whereas the use of Sulfonamides and Quinolones leads to the lowest average AMR (21.59% and 28.07%, respectively). Moreover, our analysis of antibiotic-resistant bacteria shows that *Streptococcus suis (S. suis)* and *S. auerus* provide the highest average AMR rates (71.81% and 69.48%, respectively), whereas *Campylobacter spp*. provides the lowest one (29.75%). The highest average AMR percentage, 57.46%, was observed in Mexico, followed by Canada at 45.22%, and the USA at 42.25%, which is most probably due to the presence of various AMR control strategies, such as stewardship programs and AMR surveillance bodies, existing in Canada and the USA. Our review highlights the need for better strategies and regulations to control the spread of AMR in North America.

## 1 Introduction

The increasing demand for meat around the globe has led to a significant rise in livestock breeding (Graham and Nachman, 2010; Chriki and Hocquette, 2020). Livestock are usually fed with drinkable water and food mixed with antimicrobial drugs (Sapkota et al., 2007; Brown et al., 2019). The availability and the use of antimicrobials have transmuted the practice of veterinary medicine (Lees et al., 2021; Schwarz et al., 2017; Drouillard, 2018; Prescott, 2017; Paulson et al., 2015). Several fatal animal infections have now become treatable as the antimicrobial use (AMU) has led to significant advances in global health, animal health, food safety, and food security. However, the abuse and misuse of antimicrobials have contributed significantly to the emergence and expansion of antimicrobial resistance (AMR), posing a serious threat to human and animal health as well as to the global ecosystem (Kahn, 2017; Mehrotra M, 2017; Thakur and Gray, 2019; McCubbin et al., 2021; Otto et al., 2022; Cobo-Angel and Gohar, 2022; Xu et al., 2022). Approximately, 700,000 people around the globe die every year because of antimicrobial misuse. It has been estimated that this number will increase to 10 million people by 2050 (O'Neill, 2016). According to Nathan (2020), the development of new antibiotics is declining, but the global antimicrobial consumption in food animals is accelerating. Several studies have shown that AMR of animal origin can be transmitted to humans through food production (Ribeiro et al., 2024; Martak et al., 2024) as well as to the environment (Graham et al., 2009; Fujita et al., 2022). Evidence linking AMR between animals and humans is particularly strong for common foodborne pathogens resistant to Quinolones, such as *Campylobacter spp*. and *Salmonella spp*. (Engberg et al., 2001). Nowadays, antimicrobial resistance became a major public health challenge, which requires deeper study and immediate action to combat it (World Health Organization, 2012). Van Boeckel et al. (2015) have discussed the relationships between AMU and AMR in farmed animals in a systematic review covering the period from 2000 to 2018. Following multiple international calls for urgent action, the North American countries (Canada, Mexico, and the USA) reacted to protect their population by introducing several antibiotic restriction policies discussed below.

The observation of antimicrobial use in farm animals in Canada started with the report of Health Canada in 2002 (Uses of Microbalances in Food Animals in Canada: Impact on Resistance and Human Health).^1^ The Canadian Integrated Program for Antimicrobial Resistance (CIPARS) was launched to better understand the antimicrobial resistance in livestock and its impact on human health. Since 2005, CIPARS has been publishing an annual report presenting the current situation in the field (Canadian Integrated Program for Antimicrobial Resistance Surveillance (CIPARS)).^2^ In 2014, Health Canada announced some important actions, including the strict restriction and veterinary prescription of all antimicrobial drugs. Several actors have been engaged in these actions, including the Canadian Food Inspection Agency and Agriculture and Agri-Food Canada (Antimicrobial Resistance and Use in Canada: A Federal Framework for Action).^3^ In 2017, the Canadian government started working with provincial partners to monitor antimicrobial use. Since 2018, the importation and self-manufacturing of antimicrobials have been banned in Canada. These actions were supported by the Canadian Animal Health Institute.^4^ The exact restriction policy being applied in each case differs with respect to the Canadian province, which takes full responsibility for regulatory actions. For example, in Ontario, the College of Veterinarians of Ontario, in collaboration with veterinarians and farmers, has identified the standardization of laboratory reporting as a major AMR preventing priority (College of Veterinarians of Ontario, 2017). Other associations and voluntary organizations, as for example the Canadian Cattlemen's Association, have been also involved in the establishment of the AMR restriction policies. For instance, the Canadian chicken farms have been actively involved in this work due to the spread of ceftiofur-resistant Salmonella Heidelberg pathogen (Dutil et al., 2010).

A survey conducted between 2013 and 2015 in the United States showed that 88% of veterinarians are ignorant of any veterinary professional guidelines related to AMU and AMR, thus raising the government's concern about this issue (International Society for Companion Animal Infectious Diseases).^5^ The American Food and Drug Administration (FDA) Center for Veterinary Medicine proposed some suggestions specifying the duration of AMU in food and water under veterinary oversight and providing a comprehensive AMU data collection for companion animals, thus increasing AMU data sharing. We can mention that California was the first USA state that required the use of medically important antimicrobials (Antimicrobial Use and Stewardship (Aus) Program Report to the Legislature, California, USA, 2019). Moreover, the American Veterinary Medical Association (AVMA) created an antimicrobial committee made up of the American Animal Hospital Association (AAHA) and the American Association of Swine Veterinarians (AASV) (Antimicrobial Stewardship in Companion Animal Practice, 2015). AVMA's activities involve creating and sharing guidelines as well as promoting stewardship for companion animal practice. Recently, a national veterinary regulation action plan for 2020–2025, intended to combat antibiotic-resistant bacteria and restrict the antimicrobial use in the United States, has been adopted by the Presidential Advisory Council (National Action Plan for Combating Antibiotic-Resistant Bacteria).^6^

In Mexico, a national initiative for the containment of antimicrobial resistance was endorsed by major medical, veterinary, and public health institutions to better control the situation with antimicrobial use in food-producing animals (Zaidi et al., 2015). This initiative consists in establishing of effective surveillance systems. Furthermore, the Mexican Ministry of Health issued a decree enforcing some regulations that require medical prescriptions.

Several studies have been conducted regarding the global issue of antimicrobial resistance in farm animals, and different solutions have been proposed depending on national strategies and regulations maintained by each country. In this context, we will perform a meta-analysis to identify the main AMR trends typical for the three largest North American countries Canada, Mexico, and the USA.

## 2 Methods

### 2.1 Search strategy and selection criteria

**Figure 1:**
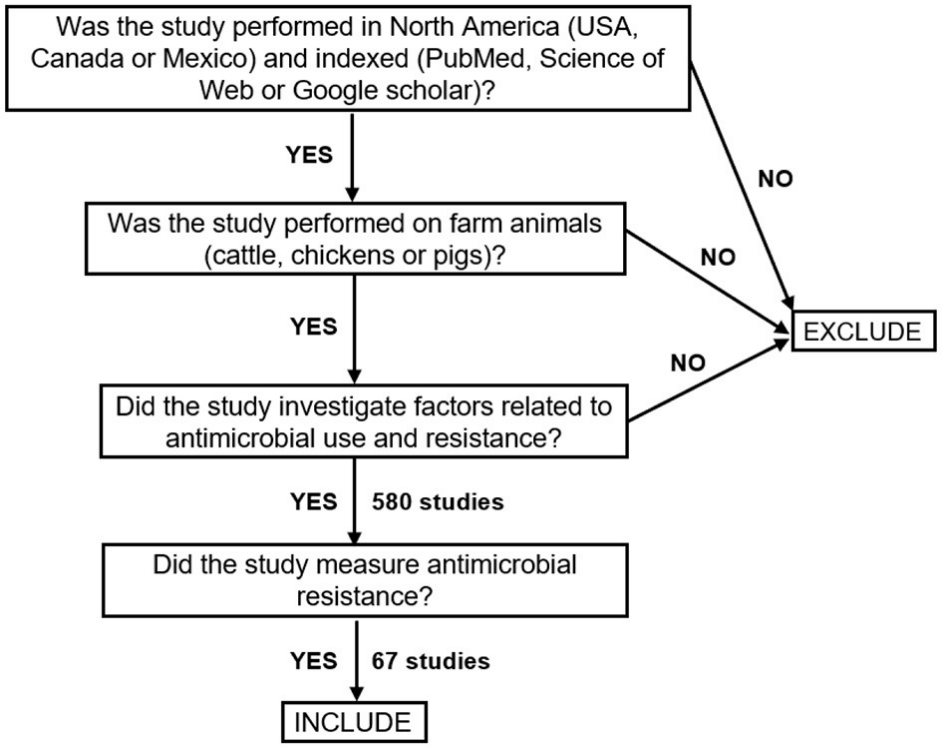
Flowchart illustrating our search selection criteria for inclusion of relevant AMR-related studies.

Google Scholar, PubMed, EMBASE, MEDLINE, Web of Science Core Collection (Science Citation Index and Emerging Sources Citation Index), and Cochrane Library have been searched to gather information on antimicrobial resistance on North American farms. Articles written in English and covering the time period from 2015 to 2024 have been selected for our review study. Search terms for our investigation included the following keywords: “antibiotic(s)”, “antimicrobial(s)”, “food animals”, “food-producing animal”, “farm animal”, “environment”, “bacteria”, “virus”, “water”, “soil”, “manure heaps”, “ponds”, “barns”, “calf hutches”, “straw and other bedding”, “feed and feed trough”, “water and water troughs”, “farm equipment”, “ground and pasture”, “watercourses, “USA”, “Canada”, “Mexico”, “cattle”, “poultry”, and “pig(s)”. The reference list of all plausible articles (published between 2015 and 2024) has been established, and the most cited articles have been considered first. In some cases, the authors, including students, professors, veterinarians, and experts in epidemiology have been contacted for some clarification about the results. The retained papers focused on the three types of food-producing animals: cattle, poultry, and pigs. As our study aims at quantifying and understanding the impact of AMR in North America, our search was limited to the studies concerning the three largest American countries: Canada, Mexico, and the USA. No search restrictions have been applied to bacterial species under study. Figure 1 shows the flowchart presenting our main search selection criteria.

### 2.2 Data analysis

Different relevant meta-data were extracted from each of the selected papers, including: Country, farm animal(s), sample type (e.g., meat or fecal matter), sampling environment (e.g., river, soil, or feedlot), living animal specimen type (e.g., swab, nasopharynges lungs and joints, blood, vaginal, paw, tissue, or saliva) or carcass specimen type (e.g., tissue or corpse). Meta-analysis has been conducted for food-producing animals only, and not for humans or the environment. Regarding food-producing animals, we limited our investigation to cattle (cow and bovine), poultry (chicken and turkey), and pigs. Regarding antimicrobials, the 11 following groups of antibiotic classes were considered: Penicillins, Tetracyclines, Sulphonamides, Macrolides, Pleuromutilins, Lincosamides, Aminoglycosides, Amphenicols, Chloramphenicol, Cephalosporins, and Quinolones.

### 2.3 Main pathways of antimicrobial resistance

**Figure 2:**
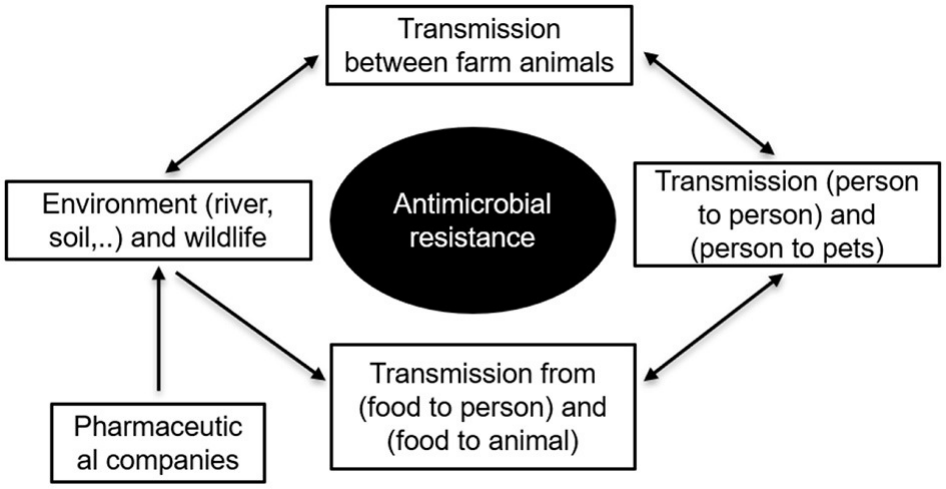
Main pathways of antimicrobial resistance spread between food-producing animals, humans, and the environment.

Minimizing the transmission of antibiotic-resistant bacteria remain a very relevant and challenging issue. Unfortunately, no universal solution has been proposed to solve it. Figure 2 presents the main pathways of antimicrobial resistance spread between animals, humans, and the environment. In many occasions, the transmission is direct, but some intermediate, often unknown, zoonotic hosts may also be involved in the chain of transmission. Direct contacts with animals can accelerate the spread of resistant bacteria as it was for example the case of the methicillin-resistant *Staphylococcus aureus (S. aureus)* bacterium isolated from the US swine population (Hau et al., 2017). Farmers, their families, and veterinarians are the most vulnerable people to be infected by antibiotic-resistant bacteria. These bacteria can be transferred to the food products at the stage of livestock slaughter as well. Obviously, humans can be also contaminated by bacteria through the meat, if it is not cooked properly (Heiman et al., 2015; Christidis et al., 2020).

Bacteria that come from animals, which can be their healthy or asymptomatic carriers, are generally pathogenic for humans, increasing the human mortality rate (Smith et al., 2020; Anomaly, 2015; Dalton et al., 2020; Rodríguez-Medina et al., 2019). Moreover, unwashed fruits or vegetables can be another path of bacterial contamination (Rahman et al., 2021; Dharmarha et al., 2019; Godínez-Oviedo et al., 2023). Vegetables can be easily contaminated through human/animal feces or wastewater (Huang et al., 2015; Huijbers et al., 2015; Ibekwe et al., 2023). The environment often plays a connection role between different farm compartments, and especially between animals compost, soil, water, sediments, and sewage. Generally, antibiotics are used for therapeutic purposes and livestock receive antibiotics in their feed for disease prevention. According to many authors, the non-therapeutic use occurs later in animals, when they reach the feedlot (Veterinary Feed Directive (VFD)).^7^ Manure is the predominant propagation pathway of AMR in farms (Dungan et al., 2018). Table 1 presents some typical examples of antibiotic resistance genes (ARGs) detected on North American farm animals, the environments, and humans. We can observe that phenotypic and molecular characterization sequencing methods, such as Polymerase Chain Reaction (PCR) and Whole Genome Sequencing (WGS), have been widely used to identify ARGs. Such a variety of studies and methods being used reveal that North American countries are very concerned with AMR detected in livestock and search for effective solutions to address this important challenge. However, the sampling and design variation makes the comparison between the resulting data fairly complicated. The global expansion of the pharmaceutical industry, driven by the rising demand for antibiotics, plays a significant role in environmental challenges. Pharmaceutical wastewater contains high concentrations of antibiotics and antibiotic resistance genes, making these areas hotspots for environmental pollution and the spread of AMR. Poor treatment and improper discharge of such wastewater into the environment result in significant antibiotic contamination, whereas its prolonged presence in the environment can alter bacterial genomes, contributing to the rise and spread of AMR (Kotwani et al., 2021).

**Table 1:** Typical examples of antimicrobial resistance genes (ARG) detected in animals, the environment, and humans (for studies conducted in Canada, the USA, and Mexico).

**Country**	**Methodology**	**AMR genes**			**Reference**
		**Animals**	**Environment**	**Humans**	
Canada	WGS	blaCMY-2, aac(3)-VIa, aac(3)-IId ant(2')-Ia, aac(6')-Ib3, ant(3”)-Ia aadA2,aadA2		blaCTX-M, blaCMY-2, aac(3)-VIa, aac(3)-IId aac(3)-Id, aac(3)IIa, ant(2”)-Ia aac(6')-Ib-cr, ant(3”)-Ia, aadA2 aadA7, aadA1, aadA17, aadA5 aadA22	(Cox et al., 2021)
	WGS	erm(B, tet(M), ant(6)-Ia, aph(3')-IIIa, sat4, tet(L), tet(M, bcrB, bcrC	erm(B), tet(M), ant(6)-Ia, aph(3')-IIIa, sat, tet(L), tet(M), ant(6)-Ia, sat4, aph(3')-IIIa		(Zaheer et al., 2020)
	qPCR		erm(A), erm(B), erm(F), erm(X), sul(1), sul(2),tet(B), tet(C), tet(H), tet(L), tet(M), tet(W)		(Holman et al., 2016)
	PCR	blaCTX-M, blaCMY-2, blaSHV, blaTEM, qnrB, qnrS			(Awosile et al., 2018)
	WGS	VmacAB, IbblaCMY-2	M,Baph(6)-Id, aph(6)-Id, tet(C) E2,J25bmrA, M,VblaCTX-M-55		(Cameron et al., 2019)
	qPCR		tet(B), tet(C), tet(L), tet(M), tet(W), erm(A),erm(B), erm(F), erm(X), sul(1), and sul(2).		(Xu et al., 2016)
	NGS	TETA, TETB, TET32, TETW, TET40, TET44, TETO, TETQ, TETX, MEFA, LNUC, APH3',ANT6, CFX, ACI	TETA, TETB, TET32, TETW, TET40, TET44, TETO, TETQ, TETX, MEFA, LNUC, APH3', ANT6, CFX, ACI, TETH, TET36, TETZ, TETS, TETT, APH6, MPHE,MPHB, MSRD ERMA, MPHE, MEL, ERMR, ERMC,		(Zaheer et al., 2019)
	PCR, WGS	blaCTX-M-55, blaCTX-M-32, blaCTX-M-27 blaCTX-M-15, blaCTX-M-14	blaCTX-M-55, blaCTX-M-32 blaCTX-M-27, blaCTX-M-15, blaCTX-M-14		(Cormier et al., 2020)
USA	WGS		blaIMP-27		(Mollenkopf et al., 2017)
	PCR		mphA, aadA, aphA1, blaTEM, tet(B), strA, penA, ampC, lde, ermB, tet(O), aadB, blaOXA-61,tet(O), and aadE		(Hailu et al., 2021)
	WGS	aac(6)-Iaa, PBR, floR, CMY, tet (A), tet (R) sul2, strA, strB, aadA, sul1, aph(3”)-Ia, tet(A) tet(R), aadA, dfrA, blaTEM-1D		aac(6)-Iaa, PBR, floR CMY, tet(A) tet(R), sul2, strA, strB, aadA, sul1 aph (3”)-Ia, aadA, dfrA, blaTEM-1D	(Carroll et al., 2017)
	WGS	blaCMY-2, blaCMY-130,
blaCMY-132,blaTEM-1A, blaTEM-1B,blaTEM-150, floR, cmlA5, qnrB19, ant(2”)-Ia, aph(3”)-Ib, aph(6)-Id (strB), aph(3')-Ia,sul1, sul2, tetA			(Srednik et al., 2021)
	PCR, PFGE		blaCMY-2		(Hsieh et al., 2016)
Mexico	PCR	tetA, tetB, strA, aadA, blaTEM, blaSHV			(Martínez-Vázquez et al., 2021)
	PCR		blaCTX-M9, blaTEM blaCTX-M151, blaCTX-M1-8 blaCTX-M-9,aac(6')-IB-cr, qepA		(Enciso-Martínez et al., 2022)
	PCR	blaCMY		blaCMY	(Aguilar-Montes de Oca et al., 2018)

## 3 Results

A total of 580 articles met the inclusion criteria mentioned above. Most of these studies discuss the use of antimicrobials and the resistance to them with respect to sample type. Table 2 presents the classification of data collections according to sample type and approach used for data analysis. The data available in this table suggest that 34% of studies have been based on the analysis of fecal or urine material used as a sample for AMR analysis. Moreover, 92% of the selected studies have been based on a laboratory analysis as an approach to detect antimicrobial resistance. We also report typical cases of antimicrobial resistance in Canada, the USA, and Mexico which were taken from the 67 articles (taken from the original list of 580 articles) that provided numerical estimates of antimicrobial resistance on North American farms. Tables 3–5, based on these 67 studies, present the AMR estimates (shown in percentages) reported for the 10 most frequent bacterial types detected and the 11 most used antibiotic classes used on North American farms. *W*e can observe that *E. coli* and *Salmonella* were the most frequent bacteria affecting North American livestock in terms of AMR, and Penicillins and Tetracyclines were among the most used antibiotic classes triggering AMR.

**Table 2:** Main sample types and approaches considered to investigate antimicrobial use and resistance on North American farms.

**Sample type**	**Approach**	**Number of studies**
Animal food (meat, milk, eggs, …)	Laboratory analysis of antimicrobial susceptibility and resistance	144
Environment (water, soil, manure, litter and feedlot)	Laboratory analysis	123
Fecal matter, urine	Laboratory analysis	197
Living animal (swab, nasopharynges lungs and joints, blood, vaginal, paw, tissue, saliva) or Carcass (tissue, corpse,…)	Laboratory analysis	71
	Discussions and in-depth observations, group discussions, and interviews	45

**Table 3:** Typical cases of antimicrobial resistance (AMR) on Canadian farms (cattle, poultry, and pigs).

**Livestock**	**Antibiotic class**	**Active ingredient**	**Bacterium**	**(%) AMR**	**Sample type**	**Reference**
Cattle	Penicillins	Ampicillin	*E. coli*	11.5	Mastitis	(Majumder et al., 2021)
		Ampicillin	*E. coli*	98.0	Manure	(Massé et al., 2023)
	Tetracylines	Tetracycline	*E. coli*	26.0	Fecal	(Massé et al., 2021)
		Oxytetracycline	*S.aureus*	96.0	Milk	(JAwosile et al., 2018)
		Doxycycline	*Enterococcus*	31.0	Fecal	(Davedow et al., 2020)
		Tetracycline	*E. coli*	15.9	Mastitis	(Majumder et al., 2021)
		Tetracyline	*E. coli*	80.0	Manure	(Massé et al., 2023)
		Tetracycline	*Salmonella*	17.0	Fecal	(Fonseca et al., 2023)
		Sulfisoxazole	*Salmonella*	13.0	Fecal	(Fonseca et al., 2023)
	Sulphonamides	Sulfisoxazole	*E. coli*	23.0	Fecal	(Massé et al., 2021)
		sulfonamide	*S.Aureus*	7.0	milk	(Naushad et al., 2020)
		Sulfisoxazole	*E. coli*	88.0	Manure	(Massé et al., 2023)
	Macrolides	Tylosin	*Enterococcus*	86.0	Fecal	(Davedow et al., 2020)
		Clindamycin	*NAS*	4.0	Milk	(Nobrega et al., 2018a)
		Erythromycin	*NAS*	100.0	Milk	(Nobrega et al., 2018b)
		Clindamycin	*NAS*	99.9	Milk	(Nobrega et al., 2018b)
		Erythromycin	*NAS*	8.0	Milk	(Nobrega et al., 2018a)
		Erythromycin	*Enterococcus*	84.0	Fecal	(Davedow et al., 2020)
	Aminoglycosides	Streptomycin	*E. coli*	19.0	Fecal	(Massé et al., 2021)
		Kanamycin	*E. coli*	15.0	Fresh colostrum	(Awosile et al., 2017)
		Streptomycin	*E. coli*	20.0	Fresh colostrum	(Awosile et al., 2017)
		Streptomycin	*E. coli*	17.7	Mastitis	(Majumder et al., 2021)
		Streptomycin	*E. coli*	73.8	Fecal	(Adator et al., 2022)
		Neomycin	*E. coli*	3.7	Fecal	(Adator et al., 2022)
		streptomycin	*Campylobacter*	3.0	Fecal	(Waldner et al., 2019)
		Streptomycin	*Salmonella*	13.0	Fecal	(Fonseca et al., 2023)
	Cephalosporins	Ceftriaxone	*E. coli*	90.0	Manure	(Massé et al., 2023)
		Ceftriaxone	*E. coli*	80.0	Fresh colostrum	(Awosile et al., 2017)
		Cephalosporin	*E. coli*	80.0	fecal	(Salaheen et al., 2019)
		Ceftiofur	*E. coli*	80.0	Fresh colostrum	(Awosile et al., 2017)
		Cefoxitin	*E. coli*	100.0	Fresh colostrum	(Awosile et al., 2017)
		Ceftiofur	*E. coli*	70.2	Manure	(Adator et al., 2022)
		Ceftiofur	*E. coli*	84.0	Manure	(Massé et al., 2023)
Poultry	Penicillins	Ampicillin	*E. coli*	16.0	Cecal	(Varga et al., 2019)
		Ampicillin	*E. coli*	44.0	Broiler	(Varga et al., 2018)
		Beta-lactam	*E. coli*	31.2	Fecal	(Shrestha et al., 2022)
	Tetracyclines	Tetracycline	*E. coli*	43.0	Fecal	(Varga et al., 2019)
		Tetracycline	*Salmonella*	42.9	Cecal	(Romero Barrios et al., 2020)
		Tetracycline	*Campylobacter*	39.0	Slaughterhouse	(Draméet al., 2020)
		Tetracycline	*Campylobacter*	48.1	Retail meats	(Narvaez-Bravo et al., 2017)
		Tetracycline	*E. coli*	57.0	Broiler	(Varga et al., 2018)
		Tetracycline	*E. coli*	61.7	Fecal	(Shrestha et al., 2022)
	Sulphonamides	Sulphonamide	*E. coli*	17.0	Cecal	(Varga et al., 2019)
		Sulfisoxazole	*Salmonella*	6.2	Cecal	(Romero Barrios et al., 2020)
		Sulfamethoxazole	*E. coli*	18.0	Broiler	(Varga et al., 2018)
	Macrolides	Azithromycin	*Campylobacter*	1.78	Retail meats	(Narvaez-Bravo et al., 2017)
		Erythromycin	*Campylobacter*	1.78	Retail meats	(Narvaez-Bravo et al., 2017)
	Aminoglycosides	Streptomycin	*E. coli*	29.0	Cecal	(Varga et al., 2019)
		Gentamicin	*Salmonella*	2.1	Cecal	(Romero Barrios et al., 2020)
		Streptomycin	*Salmonella*	41.5	Cecal	(Romero Barrios et al., 2020)
		Gentamicin	*E. coli*	50.0	Broiler	(Varga et al., 2018)
		Kanamycin	*E. coli*	11.0	Broiler	(Varga et al., 2018)
		Apramycin	*E. coli*	3.0	Broiler	(Varga et al., 2018)
		Aminoglycoside	*E. coli*	45.0	Fecal	(Shrestha et al., 2022)
	Cephalosporins	Ceftriaxone	*Salmonella*	31.4	Cecal	(Romero Barrios et al., 2020)
		Ceftiofur	*E. coli*	15.0	Broiler	(Varga et al., 2018)
	Quinolones	quinolone	*Campylobacter*	3.5	slaughterhouse	(Draméet al., 2020)
		Ciprofloxacin	*Campylobacter*	5.5	Retail meats	(Narvaez-Bravo et al., 2017)
		Nalidixic acid	*Campylobacter*	5.5	Retail meats	(Narvaez-Bravo et al., 2017)
Pigs	Penicillin	ampicillin	*E.coli*	100.0	diseased pigs	(Jahanbakhsh et al., 2016b)
		amoxicillin	*E.coli*	96.5	diseased pigs	(Jahanbakhsh et al., 2016b)
	Tetracyclines	tetracycline	*S. suis*	84.2	Nasal and vaginal swabs	(Arndt et al., 2019)
		tetracycline	*S. suis*	98.0	different sites of pigs	(Aradanas et al., 2021)
	Macrolides	Macrolide	*S. suis*	90.0	Different sites of pigs	(Aradanas et al., 2021)
	Aminoglycosides	Spectinomycin	*S. suis*	40.4	Nasal and vaginal swabs	(Arndt et al., 2019)
		Streptomycin	*E. coli*	89.4	Diseased pigs	(Jahanbakhsh et al., 2016b)
		Streptomycin	*E. coli*	91.6	Fecal	(Jahanbakhsh et al., 2016a)
		Gentamicin	*E. coli*	84.0	Diseased pigs	(Jahanbakhsh et al., 2016b)
		Kanamycin	*E. coli*	50.6	Diseased pigs	(Jahanbakhsh et al., 2016b)
	Cephalosporins	Cefoxitin	*E.coli*	96.5	Diseased pigs	(Jahanbakhsh et al., 2016a)
		Cefoxitin	*E.coli*	20.0	Diseased pigs	(Jahanbakhsh et al., 2015)
		Ceftriaxone	*E.coli*	20.0	Diseased pigs	(Jahanbakhsh et al., 2015)
		Ceftiofur	*E.coli*	20.0	Diseased pigs	(Jahanbakhsh et al., 2015)
		Ceftiofur	*E.coli*	100.0	Diseased pigs	(Jahanbakhsh et al., 2016b)
	Quinolone	Nalidixic acid	*E.coli*	4.7	Different sites of pigs	(Jahanbakhsh et al., 2016b)

**Table 4:** Typical cases of antimicrobial resistance (AMR) on the US farms (cattle, poultry, and pigs).

**Livestock**	**Antibiotic class**	**Active ingredient**	**Bacterium**	**(%) AMR**	**Sample type**	**Reference**
Cattle	Penicillins	beta lactam	*Salmonella*	85.0	Living animal and fecal	(Srednik et al., 2021)
		Penciilin	*S. areaus*	63.2	Beef	(Rao et al., 2021)
		Ampicillin	*E. coli*	100.0	Fecal	(Morris et al., 2023)
		Ampicillin	*Enterococcus*	0.4	Fecal	(Morris et al., 2023)
	Tetracyclines	Tetracycline	*E. coli*	27.5	Fecal	(Jeamsripong et al., 2021)
		tetracycline	*Campylobacter*	88.1	Feedlot	(Tang et al., 2017b)
		tetracycline	*E. coli*	74.8	Feedlot	(Tang et al., 2017b)
		tetracycline	*Salmonella*	21.74	Fecal	(Dargatz et al., 2016)
	Sulphonamides	sulphonamide	*E. coli*	22.5	Fecal	(Jeamsripong et al., 2021)
		sulphadimethoxine	*S.aureus*	25.0	Milk	(Abdi et al., 2018)
		sulphadimethoxine	*E. coli*	32.4	Fecal	(Abdelfattah et al., 2021)
		sulfadimethoxine	*E. coli*	25.4	fecal	(Morris et al., 2023)
		Sulfisoxazole	*Salmonella*	12.4	Fecal	(Dargatz et al., 2016)
	Macrolides	Macrolide	*Enterococcus*	77.6	Fecal	(Jeamsripong et al., 2021)
		Macrolide	*Salmonella*	77.0	Manure	(Hailu et al., 2021)
		Macrolide	*E.coli*	73.0	Manure	(Hailu et al., 2021)
		Erythromycin	*S. areaus*	15.8	Beef	(Rao et al., 2021)
		Erythromycin	*Enterococcus*	14.9	Retrail	(Tate et al., 2021)
		Azithromycin	*C. jejuni*	0.3	Feedlot	(Tang et al., 2017b)
		Azithromycin	*E.coli*	0.04	Fecal	(Levent et al., 2022)
		Clindamycin	*C. jejuni*	0.3	Feedlot	(Tang et al., 2017b)
		Clindamycin	*C. coli*	4.3	Feedlot	(Tang et al., 2017b)
		Azithromycin	*C. coli*	0.0	Feedlot	(Tang et al., 2017b)
		Erythromycin	*C. jejuni*	0.3	Feedlot	(Tang et al., 2017b)
	Aminoglycosides	Aminoglycoside	*Salmonella*	95.0	Living animal and fecal	(Srednik et al., 2021)
		Streptomycin	*E. coli*	49.45	Retrail	(Tate et al., 2021)
		Aminoglycoside	*E. coli*	7.2	Fecal	(Doster et al., 2022)
		Streptomycin	*Enterococcus*	19.0	Retrail	(Tate et al., 2021)
	Cephalosporins	Ceftiofur	*E. coli*	0.4	Fecal	(Morris et al., 2023)
	Quinolones	ciprofloxacin	*C. jejuni*	35.6	Feedlot Cattle	(Tang et al., 2017b)
		Quinolone	*C. coli*	60.0	Retail meat	(Hull et al., 2021)
		Ciprofloxacin	*C. coli*	74.4	Feedlot cattle	(Tang et al., 2017b)
		Nalidixic acid	*C. jejuni*	34.3	Feedlot cattle	(Tang et al., 2017b)
		Nalidixic acid	*C. coli*	82.6	Feedlot cattle	(Tang et al., 2017b)
Poultry	Penicillin	Penicillin	*C. coli*	63.6	Fresh poultry products	(Hull et al., 2021)
	Tetracyclines	Tetracycline	*Salmonella*	63.0	Cloacal swabs	(Velasquez et al., 2018)
		Tetracycline	*C. coli*	64.3	Fresh poultry products	(Hull et al., 2021)
		Tetracycline	*Salmonella*	13.9	Environment and carcasses	(Liljebjelke et al., 2017)
		Tetracycline	*Salmonella*	76.0	broiler farm	(Rama et al., 2022)
		Tetracycline	*Salmonella*	52.17	Retail chicken	(Lee et al., 2018)
	Macrolides	Macrolide	*C. coli*	34.8	Cloacal swabs	(Velasquez et al., 2018)
	Aminoglycosides	Aminoglycoside	*C. coli*	38.6	Fresh poultry products	(Hull et al., 2021)
		Streptomycin	*Salmonella*	30.9	Environment and carcasses	(Liljebjelke et al., 2017)
		Gentamicin	*Salmonella*	12.6	Environment and carcasses	(Liljebjelke et al., 2017)
		Streptomycin	*Salmonella*	70.0	Broiler farm	(Rama et al., 2022)
		Streptomycin	*Salmonella*	52.17	Retail chicken	(Lee et al., 2018)
	Quinolones	nalidixic acid	*Salmonella*	5.0	Cloacal swabs	(Velasquez et al., 2018)
		Quinolone	*C. coli*	24.4	Fresh poultry products	(Hull et al., 2021)
Pigs	Pencillin	Ampicillin	*E. coli*	21.1	cecal	(Sodagari and Varga, 2023)
		penicillin	*S. aureus*	72.0	Fecal	(Beier et al., 2021)
	Tetracyclines	Tetracycline	*Salmonella*	57.6	Manure and soil	(Pornsukarom and Thakur, 2016)
		tetracycline	*S. suis*	97.0	Swine	(Nicholson and Bayles, 2022)
		Tetracycline	*E. coli*	65.3	Cecal	(Sodagari and Varga, 2023)
		Tetracycline	*S. aureus*	50.0	Pig pens	(Randad et al., 2021)
	Sulphonamides	Sulfisoxazole	*Salmonella*	67.2	Manure and soil	(Pornsukarom and Thakur, 2016)
	Macrolides	Tilmicosin	*S. aureus*	78.5	Nasal swabs and environment	(Hau et al., 2018)
		Macrolide	*S. suis*	72.0	Swine	(Nicholson and Bayles, 2022)
		Macrolide	*S. aureus*	56.0	Pig pens	(Randad et al., 2021)
	Aminoglycosides	Streptomycin	*Salmonella*	88.3	Manure and soil	(Pornsukarom and Thakur, 2016)
		Streptomycin	*E. coli*	20.4	Cecal	(Sodagari and Varga, 2023)
		Aminoglycoside	*S. aureus*	62.0	Pig pens	(Randad et al., 2021)
	Cephalosporins	Ceftiofur	*E. coli*	82.1	Diseased pigs	(Hayer et al., 2020b)
		Ceftiofur	*E. coli*	34.1	Diseased pigs	(Hayer et al., 2020a)
	Quinolones	Enrofloxacin	*E. coli*	81.8	Diseased pigs	(Hayer et al., 2020b)
		Quinolone	*S. suis*	21.8	Pig	(Hayer et al., 2020b)
		Quinolone	*Salmonella*	10.1	Pig	(Pires et al., 2021)

**Table 5:** Typical cases of antimicrobial resistance (AMR) on Mexican farms (cattle, poultry, and pigs).

**Livestock**	**Antibiotic class**	**Active ingredient**	**Bacterium**	**(%) AMR**	**Sample type**	**Reference**
Cattle	Penicillins	Penicillin G	*S. aureus*	36.8	Composite milk and hand swabs	(Salgado-Ruiz et al., 2015)
		ampicillin	*S. aureus*	83.3	milk	(Guzmán-Rodríguez et al., 2021)
		Oxacillin	*S. aureus*	36.6	milk	(Guzmán-Rodríguez et al., 2021)
		Penicillin	*S. aureus*	100.0	Milk	(Varela-Ortiz et al., 2018)
		Ampicillin	*Salmonella*	40.9	Fecal, carcass, cut and ground beef	(Delgado-Suárez et al., 2019)
		Carbenicillin	*Salmonella*	29.5	Fecal, carcass, cut and ground beef	(Delgado-Suárez et al., 2019)
		Benzylpenicillin	*S. aureus*	97.0	milk	(Mora-Hernández et al., 2021)
		Ampicillin	*E. coli*	94.0	Irrigation water, harvesting melons, hands of workers. and boxes	(Enciso-Martínez et al., 2022)
		Ampicillin	*E. coli*	83.0	Feces and carcass	(Martínez-Vázquez et al., 2021)
		Ampicillin	*Salmonella*	94.73	Retail beef	(Nova Nayarit-Ballesteros et al., 2016)
		Carbenicillin	*Salmonella*	84.21	Retail beef	(Nova Nayarit-Ballesteros et al., 2016)
	Tetracyclines	Tetracycline	*S. aureus*	77.0	Cow Milk	(Varela-Ortiz et al., 2018)
		Tetracycline	*Salmonella*	90.9	Fecal, carcass, cut and ground beef	(Delgado-Suárez et al., 2019)
		tetracycline	*E. coli*	69.0	Fecal, carcass, cut and ground beef	(Martínez-Vázquez et al., 2021)
		Tetracycline	*Salmonella*	40.2	Fecal	(Maradiaga et al., 2019)
		Tetracycline	*E. coli*	86.6	Fecal	(Mandujano et al., 2023)
		Tetracycline	*Salmonella*	68.42	Retail beef	(Nova Nayarit-Ballesteros et al., 2016)
	Aminoglycosides	Streptomycin	*Salmonella*	36.3	Fecal, carcass, cut and ground beef	(Delgado-Suárez et al., 2019)
		Streptomycin	*E.coli*	83.3	Fecal	(Mandujano et al., 2023)
		Aminoglycoside	*Salmonella*	7.8	Lymph nodes	(Delgado-Suárez et al., 2021)
		Gentamicin	*E. coli*	93.3	Fecal	(Mandujano et al., 2023)
	Cephalosporins	Cephalothin	*S. aureus*	100.0	cow Milk	(Varela-Ortiz et al., 2018)
		Cephalothin	*E. coli*	76.0	Feces and carcass	(Martínez-Vázquez et al., 2021)
		Cefotaxime	*S. aureus*	86.6	Milk	(Guzmán-Rodríguez et al., 2021)
		ceftazidime	*S. aureus*	80.0	Milk	(Guzmán-Rodríguez et al., 2021)
		Ceftazidime	*E. coli*	1.3	Carcasse	(Aguilar-Montes de Oca et al., 2015)
	Quinolones	Nalidixic acid	*E. coli*	64.0	Carcasse	(Aguilar-Montes de Oca et al., 2015)
		Ciprofloxacin	*E. coli*	10.0	carcasse	(Aguilar-Montes de Oca et al., 2015)
		Nalidixic acid	*Salmonella*	21.1	Fecal	(Maradiaga et al., 2019)
Poultry	Penicillins	Ampicillin	*E. coli*	80.7	cloacal swab	(Talavera-González et al., 2021)
		Carbenicillin	*E. coli*	56.3	Cloacal swab	(Talavera-González et al., 2021)
		Carbenicillin	*Salmonella*	26.0	Ground beef	(Delgado-Suárez et al., 2021)
		Amoxicillin-clavulanic acid	*Salmonella*	20.8	Ground beef	(Delgado-Suárez et al., 2021)
	Tetracyclines	Tetracycline	*E. coli*	64.4	cloacal swab	(Talavera-González et al., 2021)
		Tetracycline	*Salmonella*	40.3	Ground beef	(Delgado-Suárez et al., 2021)
	Aminoglycosides	amikacin	*Enterococcus*	42.0	Chicken	(almada Corral et al., 2023)
		Kanamycin	*Enterococcus*	38.0	chiken	(almada Corral et al., 2023)
		Streptomycin	*Enterococcus*	55.0	Chiken	(almada Corral et al., 2023)
	Macrolides	Erythromycin	*Enterococcus*	33.0	Chiken	(almada Corral et al., 2023)
	Quinolones	Nalidixic acid	*E. coli*	26.9	Cloacal swab	(Talavera-González et al., 2021)
		Ciprofloxacin	*Salmonella*	26.0	Ground beef	(Delgado-Suárez et al., 2021)
Pigs	Penicillins	Penicillin	*E. coli*	92.0	meat	(Martínez-Vázquez et al., 2018)
		Ampicillin	*S. aureus*	85.0	Meat	(Martínez-Vázquez et al., 2021)
		Penicillin	*S. aureus*	86.2	Meat	(Martínez-Vázquez et al., 2021)
		Penicillin	*E. coli*	44.8	Water	(Canizalez-Roman et al., 2019)
	Tetracyclines	Tetracycline	*E. coli*	75.0	Meat	(Martínez-Vázquez et al., 2018)
		Tetracycline	*E. coli*	37.9	Water	(Canizalez-Roman et al., 2019)
		Tetracycline	*Salmonella*	73.7	Slaughterhouse	(Vega-Sánchez et al., 2020)
	Sulphonamides	Sulfamethoxazole-trimethoprim	*E. coli*	13.8	Water	(Canizalez-Roman et al., 2019)
	Aminoglycosides	Gentamicin	*E. coli*	6.9	Water	(Canizalez-Roman et al., 2019)
		Aminoglycoside	*Salmonella*	100.0	Slaughterhouse	(Vega-Sánchez et al., 2020)
	Cephalosporins	Cefazolin	*E. coli*	92.0	Meat	(Martínez-Vázquez et al., 2018)
		Cefotaxime	*E. coli*	78.0	Meat	(Martínez-Vázquez et al., 2018)
	Quinolones	Ciprofloxacin	*E. coli*	3.5	Water	(Canizalez-Roman et al., 2019)
		Nalidixic acid	*E. coli*	3.5	Water	(Canizalez-Roman et al., 2019)
		Ciprofloxacin	*Salmonella*	44.7	Slaughterhouse	(Vega-Sánchez et al., 2020)

**Figure 3:**
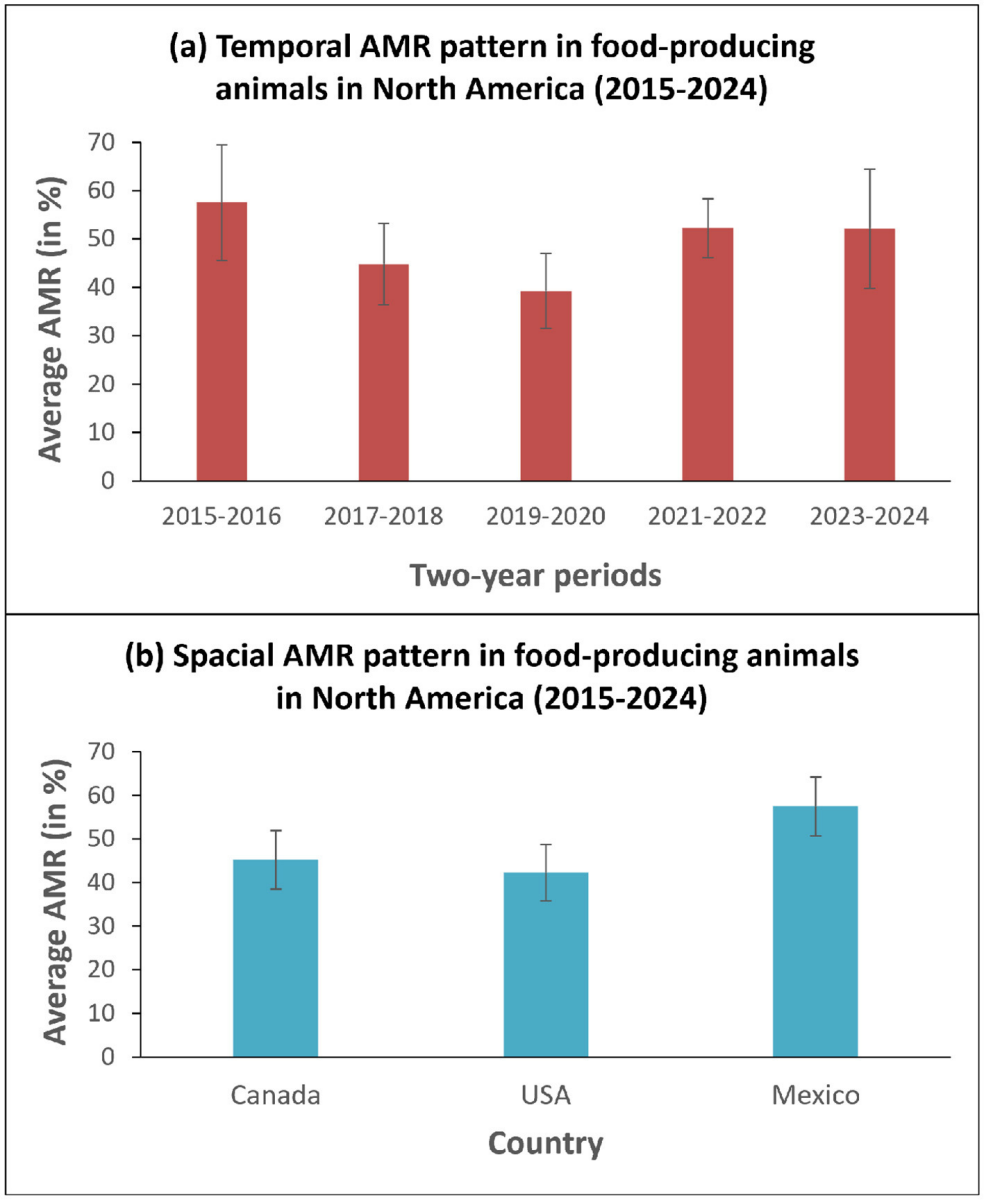
Average AMR rates on: **(a)** the temporal (by a two-year period within 2015–2024) and **(b)** the spatial (by North American country) scales. The associated 90% confidence intervals (CI) are shown.

**Figure 4:**
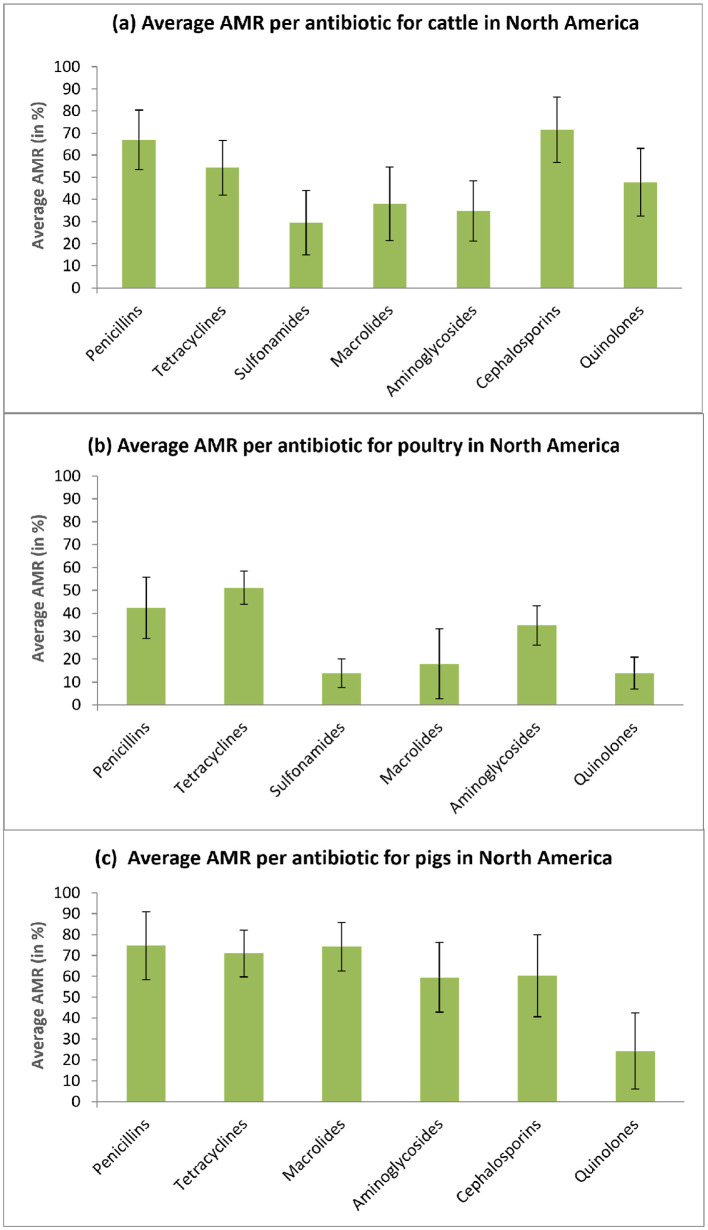
Average AMR per antibiotic class rates for cattle, poultry, and pig farms in North America. The associated 90% confidence intervals are reported. **(a)** Average AMR per antibiotic for cattle in North America. **(b)** Average AMR per antibiotic for poultry in North America. **(c)** Average AMR per antibiotic for pigs in North America.

**Figure 5:**
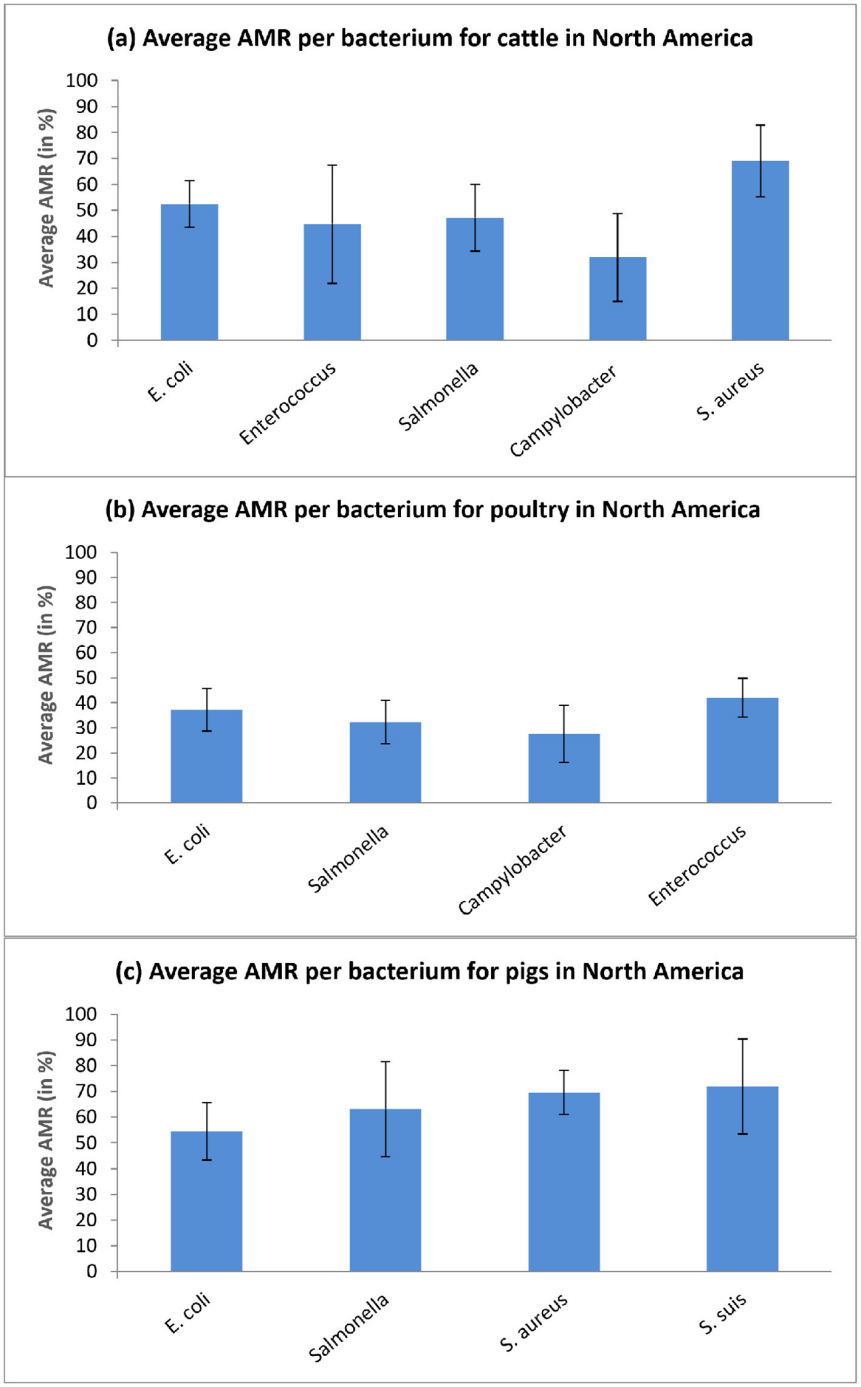
Average AMR rates per bacterium for cattle, poultry, and pig farms in North America. The associated 90% confidence intervals are reported. **(a)** Average AMR per bacterium for cattle in North America. **(b)** Average AMR per bacterium for poultry in North America. **(c)** Average AMR per bacterium for pigs in North America.

Comparing the average AMR rates across the three largest North American countries (see Tables 3–6 as well as Figures 3–**5**), one can observe the following trends: Regarding cattle, the USA have the lowest average AMR rate of 35.67%, followed by Canada with the average AMR rate of 49.60%, and Mexico with the highest average AMR rate of 64.45%. In contrast, in pig farming, Canada shows the highest average AMR rate of 67.86%, compared to Mexico with 55.80%, and the USA with 57.62%. Finally, for poultry, Canada shows the lowest average AMR rate at 25.31%, while the USA and Mexico have much higher average AMR rates of 42.96% and 42.45%, respectively. The related confidence intervals of the observed AMR cases are generally much longer for cattle than for pigs, and especially than for poultry that provide the lowest estimates. Obviously, the observed AMR rate depends highly on the antibiotic type being used and the bacterium being treated.

**Table 6:** Average AMR rates (in %) and the corresponding standard deviations (STD) obtained for food-producing animals in North America during the 2015–2024 time period.

	**Livestock**		**Average**	**STD**	**CI**
Antibiotics	Cattle	Penicillins	66.94	33.66	13.42
		Tetracyclines	54.30	30.78	12.28
		Sulfonamides	29.46	24.95	14.50
		Macrolides	37.96	41.66	16.62
		Aminoglycosides	34.78	33.14	13.63
		Cephalosporins	71.42	32.46	14.80
		Quinolones	42.32	26.19	15.23
	Poultry	Penicillins	42.32	22.98	13.31
		Tetracyclines	51.21	15.94	7.27
		Sulfonamides	13.73	6.54	6.21
		Macrolides	17.84	18.55	15.26
		Aminoglycosides	34.72	19.98	8.84
		Quinolones	13.82	11.21	6.96
	Pigs	Penicillins	74.70	27.84	16.19
		Tetracyclines	70.96	20.46	11.12
		Macrolides	74.12	14.19	11.67
		Aminoglycosides	59.54	33.56	16.64
		Cephalosporins	60.30	35.77	19.6
Bacteria	Cattle	Quinolones	24.30	29.38	18.26
		*E. coli*	52.14	34.90	8.96
		*Enterococcus*	44.70	36.58	22.74
		*Salmonella*	47.12	32.94	12.77
		*Campylobacter*	31.93	35.61	16.91
		*S. aureus*	69.02	30.30	13.82
	Poultry	*E. coli*	37.17	21.79	8.45
		*Salmonella*	32.28	23.11	8.72
		*Campylobacter*	27.57	23.86	11.33
		*Enterococcus*	42.00	9.41	7.74
	Pigs	*E. coli*	54.48	35.89	11.15
		*Salmonella*	63.08	29.73	18.48
		*S. aureus*	69.55	13.78	8.56
		*S. suis*	71.91	29.69	18.46

Confidence intervals (CI) computed for the alpha parameter of 0.1 (i.e. 90%) are reported.

Furthermore, we conducted a detailed analysis to compare separately, for cattle, poultry, and pigs raised on North American farms, the average AMR rates per antibiotic class (Figure 4) and per bacterium being treated (see Figure 5). A 90% confidence interval (CI) was calculated for each AMR estimate considered.

Figure 4 illustrates the average AMR rates for each antibiotic class in cattle (a), poultry (b), and pigs (c) farms across North America. For cattle, the highest value of antibiotic resistance is observed with Cephalosporins – 71.42% on average [90% CI: 56.62% to 86.22%], for poultry, the highest AMR is observed with Tetracyclines – 51.21% on average [90% CI: 43.94% to 58,48%], while for pigs, the highest AMR is found with Penicillins - 74.7% on average [90% CI: 58.51% to 90.89%].

Figure 5 shows the average AMR rates per bacterium, characteristic for the cattle (a), poultry (b), and pig (c) farms in North America. The highest AMR for cattle is found with *S. areaus* – 69.02% on average [90% CI: 55.2% to 82.84%], for poultry, the highest AMR percentage is found with *Enterococcus* – 42.0% on average [90% CI: 34.26% to 49.74%], while for pigs, the highest AMR is observed with *Streptococcus suis (S. suis)* – 71.91% on average [90% CI: 53.45% to 90.37%].

Moreover, we determined that the highest average AMR rates have been observed for pigs – 60.63%, on average, the medium for cattle – 48.94%, on average, and the lowest for poultry – 28.43%, on average. The presented results indicate that Cephalosporines, Penicillins, and Tetracyclines are the antibiotic classes with the highest average AMR rate – 65.86%, 61.32%, and 58.82%, respectively, whereas the use of Sulfonamides and Quinolones leads to the lowest average AMR – 21.59% and 28.07%, respectively. Regarding antibiotic-resistant bacteria, we found that *S. suis* and *S. auerus* provide the highest average AMR rates – 71.81% and 69.48%, respectively, while *Campylobacter spp*. provides the lowest average AMR of 29.75%.

Table 6 reports the average AMR rates along with the corresponding standard deviations (STD) and 90% confidence intervals (CI) obtained for the 2015–2024 time period. The most important AMR percentage variations are observed for Cattle, followed by Pigs, and then by Poultry that correspond to the lowest AMR scores and STD values.

Figure 3a presents the average AMRs over two-year periods (from 2015 to 2024) in North American farms. The averages were calculated over all types of livestock and antimicrobials considered. We can observe an important trend consisting in the decrease of an average AMR from 57.5 % in 2015–2016 to 39.25% in 2019–2020. However, this trend was reversed in 2021–2022 and 2023–2024 as the AMR rate increased again, reaching the level of 52%. Figure 3b illustrates the spatial AMR pattern characteristic for North American food-producing animal farms. The highest average AMR percentage, 57.46%, was observed in Mexico, followed by Canada at 45.22%, and the USA at 42.25%. This trend can be explained by a better AMR control, carried out through different programs and strategies discussed above, existing in Canada and the USA, compared to Mexico.

## 4 Discussion

Nowadays, the use of antibiotics on farms to prevent bacterial propagation is a topic of discussion around the world (Holmes et al., 2016; Wu et al., 2023; Galiot et al., 2023; Mohsin et al., 2023; Gao et al., 2023). Obviously, antibiotics given to livestock have the benefits of treating, reducing, and preventing bacterial infections. However, the downside is also evident. Antibiotics impose strong selective pressure on microbial populations so that their excessive use in food-producing animals makes the targeted bacteria not only resistant to antimicrobials but also transferable to humans, thus contributing to the emergence of new antibiotic-resistant human pathogens (e.g., antibiotic-resistant bacteria can be easily transferred to humans through the consumption of meat, fruits, or vegetables) (O'Neill, 2016; Manyi-Loh et al., 2018; Salam et al., 2023; Kaur et al., 2024).

Several studies have suggested that AMU in animals can bring resistance to various zoonotic pathogens (Dutil et al., 2010; Rhouma et al., 2021; Innes et al., 2020; Huber et al., 2021; Ekakoro et al., 2019; Léger et al., 2022). Moreover, the transmission of antibiotic-resistant bacteria can go in the opposite direction, i.e., from humans to animals. This kind of transmission is much less studied, however. Some recent works in the field have been devoted to the investigation of different cases of human-to-animal transmission of antimicrobial resistance, involving pets (Haenni et al., 2012; Redding et al., 2023; Roken et al., 2022) and livestock (Khanna et al., 2008). Yet, AMR can be transmitted from humans to animals, and then be back to humans. Weese et al. indicated the presence of human clones of methicillin-resistant *S. aureus* in horses (Weese et al., 2005). Dierikx et al. discussed the presence of common human clones of multidrug-resistant *Enterococcus* in pets (Dierikx et al., 2012). Several recent studies argued that AMU in different animals can contribute to AMR to several animal pathogens (Beck et al., 2012; Agunos et al., 2019; Pinto et al., 2023; Ida et al., 2023; Ekhlas et al., 2023). A number of recent studies discussed the facts of transmission of antimicrobial resistance genes from animals to soil pathogens through manure and wastewater irrigation (Sancheza et al., 2016; Williams-Nguyen et al., 2016; Scott et al., 2018; Murray et al., 2019; Mays et al., 2021). For instance, antimicrobial resistance genes in surface and groundwater can propagate to indigenous organisms through horizontal gene transfer (HGT) (Boc and Makarenkov, 2011; Gou et al., 2018; Makarenkov et al., 2021). According to Dungan et al. (2018), manure containing antibiotic resistance genes (ARGs) is the most important propagation pathway in the environment. According to Qian et al. (2018), pig and chicken manures show a greater ARG diversity than cow manures. This can be explained by the fact that over their lifetime pigs and chickens usually receive a higher dosage of antibiotics than cows (Dunlop et al., 1998; Dewey, 1999). However, no qualitative or quantitative studies have been conducted so far to explain in detail the relationships between AMU and the emergence of ARG.

Today, many public and governmental organizations in North America continue to argue for reducing the use of antibiotics in livestock. According to Moreno (2012), a referendum involving 1,000 US residents showed that 72% of them were apprehensive about the excess of antibiotics in animal feed. In 2012, the US Food and Drug Administration forbade unapproved doses of cephalosporins (Cephalosporin Order of Prohibition Goes Into Effect).^8^ Still in 2012, Barbara Sibbald (Deputy Editor of Canadian Medical Association journal) raised a danger alert for stricter regulations on antibiotic use in farm animals in Canada (Sibbald, 2012). In the province of Quebec (Canada), the resistance of porcine *Escherichia coli (E. coli)* isolates to ceftiofur has increased from 0% in 1994 to 20% in 2011 (Surveillance de l'antibiorésistance-Rapport Annuel, 2011). Moreover, according to Park et al. (2013), 97% of the *Staphylococcus hyicus* isolates from pigs in the province of Ontario (Canada) have been resistant to penicillin G and ampicillin, whereas 71% of these isolates have been resistant to ceftiofur. A study conducted in the USA in 2023 revealed that *Salmonella* found in American poultry show a high resistance (73.1% on average) to multiple antibiotics, including fluoroquinolones and extended-spectrum cephalosporins. It poses a significant public health concern as these antibiotics are also commonly used to treat *Salmonella* infections in humans (Mujahid et al., 2023). Besides the commonly discussed antibiotics, antimicrobial resistance to pleuromutilins (Hayer et al., 2020a), lincosamides (Abdelfattah et al., 2021), amphenicols (Nobrega et al., 2018b), and chloramphenicols (Vounba et al., 2019) has also been observed in food-producing animals across North America. Although these antibiotics have been used less frequently, they still contribute to a broader issue of the AMR spread in livestock.

Certainly, the use of antibiotics in livestock around the world needs to be better analyzed and characterized by conducting new quantitative or qualitative studies and surveys as it has been recently done by Kimera et al. (2020) in an African perspective. Real-life data should be made available to allow decision-makers to know where we currently stand. This kind of studies can be used not only to compare the AMU and AMR relationships in different countries, but also to take action and help reduce unnecessary antimicrobial use. Denmark and the Netherlands are examples of countries that applied different AMR prevention approaches to reduce antibiotic usage in farm animals (Aarestrup et al., 2010). For example, by 2008 in Denmark, pig production was using less than 50% of antibiotics of the total they were using in 1992. The Netherlands launched, in 2009, a project intended to minimize the antibiotic use by 50% in three years. The proposed measures helped reduce sales of antibiotics in the Netherlands by 32% (Trends in Veterinary Antibiotic Use in the Netherlands 2005-2011, 2011). Since 2020, Norway has been implementing a cyclical approach to combat antimicrobial resistance based on a new national strategy (Rortveit and Simonsen, 2020). Rørtveit and Simonsen explored the key elements and the effectiveness of this approach, and described primary care perspective on the Norwegian national strategy against antimicrobial resistance (Rortveit and Simonsen, 2020).

The spread of antimicrobial resistant genes in livestock and their transfer to humans become more and more challenging issues not only in North America, but in many countries around the globe. The need for understanding how to reduce the transmission of ARG from food-producing animals to humans has become a topic of major importance.

To effectively address the potential health risks related to AMR, it is crucial to adopt the OneHealth approach (Asaaga et al., 2022) that highlights the need for collaboration between human, animal, and environmental health sectors to effectively mitigate AMR risks. This approach aims at tackling AMR by encouraging global collaboration, innovation, and accountability. It includes using antibiotics only when necessary for treatment, avoiding their use as growth promoters, and regulating their use in both humans and animals. By offering a comprehensive strategy, the OneHealth framework promotes stronger global governance, sustainable practices, and monitoring to control the spread of resistant bacteria. This integrated approach is essential to reduce AMR risks and ensure long-term health for all.

Some specific approaches have already shown their effectiveness in reducing the use of AMR in farms (Aarestrup et al., 2010; Trends in Veterinary Antibiotic Use in the Netherlands 2005-2011, 2011; Rortveit and Simonsen, 2020). They include creating a private place for infected animals, minimizing contacts between humans and animals, and optimizing waste collection. A deep understanding of the mechanisms related to animal maintenance is fundamental for understanding how livestock waste can accelerate the spread of AMR. Obviously, farmers should pay particular attention when they use antibiotics because not all bacterial infections need antibiotic treatment (Ventola, 2015). Thus, the increase in the number of sick animals on a farm is not a cause for antimicrobial misuse. Sometimes, inflammatory conditions, such as pancreatitis or neoplasia are considered and treated as bacterial infections. In many cases, incision and drainage represent an alternative for treatment of localized abscesses. Prevention in early times can also help heal a secondary bacterial infection without antimicrobials (Lhermie et al., 2018).

Spatio-temporal factors provide important information that can help policymakers, researchers, and veterinarians take action to reduce or prevent the spread of AMR (Asaduzzaman et al., 2022). For instance, a study by Sodagari et al. revealed higher levels of antimicrobial resistance in *Escherichia coli* isolates from eggs produced in cage-free systems compared to cage systems, particularly after the tetracycline and amoxicillin treatment (Sodagari et al., 2023). A study conducted by Novoa Rama examined the impact of housing systems on the prevalence and AMR of *Campylobacter jejuni (C. jejuni)*. The results showed a higher prevalence of bacteria in hens from cage-free systems, with high resistance to tetracycline (67%) (Novoa Rama et al., 2018). These findings highlight the significance of housing systems as an environmental factor in the spatial distribution of AMR. In addition, the rearing period of animals plays a role in AMR development. Montoro-Dasi et al. (2020) compared two breeds of hens–one with rapid growth and the other with slow growth. The results suggest that fast-growing hens had higher AMR rates at the beginning of their rearing period. However, by the end of the growth period, no significant difference was observed between the two groups, indicating that AMR can develop rapidly under certain production conditions, even without antibiotic use. Thus, AMR dynamics are obviously influenced by both environmental and temporal factors, which should be considered when developing strategies to reduce AMR spread. This understanding can help inform more targeted intervention efforts and policies aimed at controlling AMR in agricultural settings.

AMU data can be considered as well to explore the relationship between the use of antimicrobials and the emergence of antimicrobial resistance to specific bacterial strains (Holmer et al., 2019). For example, the administration of ceftiofur in-ovo or to day-old chicks in hatcheries was associated with the emergence of the ceftiofur resistance in Salmonella Heidelberg found in the chicken meat. A noticeable reduction of this kind of AMR was observed when hatcheries in Quebec voluntarily ceased the in-ovo use of ceftiofur (Dutil et al., 2010). It has been shown that infections caused by antibiotic-resistant *Campylobacter* strains may lead to more frequent and prolonged hospitalizations compared to infections caused by non-resistant strains (Igwaran and Okoh, 2019). *Campylobacter* is one of the main source of bacterial foodborne and waterborne infections, including diarrhoeal diseases. Although most pig herds carry *Campylobacter coli (C. coli)*, limited research has explored the relationship between AMU and AMR to *Campylobacter* in pigs (Tang et al., 2017a). This gap may be attributed to the prevailing focus on poultry as a primary source of human exposure to *Campylobacter* (Igwaran and Okoh, 2019). While campylobacteriosis is a less common cause of clinical illness in pigs, they still pose a potential risk for foodborne campylobacteriosis, environmental contamination, and exposure of farm workers to Campylobacter.

It is worth noting that the decrease in antibiotic use does not always decrease AMR. For example, Borgen et al. (2000) observed the persistence of vancomycin-resistant *Enterococcus* on Norwegian poultry farms even after the prohibition of the avoparcin. According to Lopatkin et al. (2017), the conjugation of plasmids carrying an antimicrobial gene can result in plasmid maintenance in a microbial community in the absence of antibiotics. It is imperative to embrace a fresh perspective that not only aims at decreasing antibiotic usage but also focuses on preventing the unification of resistance as well as on promoting the preservation of plasmids. Several alternatives to combat antimicrobial resistance have been proposed in the literature, including prebiotics (Hume, 2011; Cunningham et al., 2021; Yang et al., 2019), antimicrobial peptides (Rima et al., 2021; rudzynski K, 2015), and probiotics (Yaqoob et al., 2022; Lone et al., 2022). For example, prebiotics can help modify the animal's gut by regulating its immune systems (Pourabedin and Zhao, 2015). Ghosh et al. (2019) discussed the current state of these alternatives and highlighted the main difficulties of their implementation.

Furthermore, some strategies should be implemented to limit the transmission of antimicrobial resistance through the environment. For example, some preventive measures must be applied to manure storage and disposal. Farm workers should pay attention to conventional waste treatment. Disinfection with chlorine is a fundamental step to treat the wastewater on farms (Yuan et al., 2015). In addition, farmers need to stop applying livestock waste-amended manure to soils to prevent the transfer of AMR from soil pathogens to humans (National Antimicrobial Resistance Monitoring System (NARMS)).^9^

## 5 Conclusion

Food-producing animals have been identified as a significant contributor to the dissemination of antimicrobial resistance as indicated by the high levels of AMR observed in livestock in the three largest North American countries. High AMR rates, observed especially for cattle and pigs, can be a cause of transmission of AMR to humans who come in contact with farm animals, directly or indirectly through contaminated food products or the environment.

Our review only touches the surface of a vast global issue, which requires urgent attention and coordinated efforts of farmers and veterinarians. It is important to note that our study was not designed to recommend any specific type or level of restriction on antibiotic use. Our research is rather focused on quantifying and comparing AMR rates in food-producing animals, including cattle, poultry, and pigs, in North America. We reviewed the proposed solutions to combat AMR in the three largest North American countries and suggested some complementary strategies which could aid to reduce antimicrobial resistance in livestock. Our findings can be used to develop new policies and approaches to address this pressing global concern.

## Data Availability

The original contributions presented in the study are included in the article/supplementary material, further inquiries can be directed to the corresponding author.

## Footnotes

^1^https://www.canada.ca/en/health-canada/services/drugs-health-products/reports-publications/veterinary-drugs/uses-antimicrobials-food-animals-canada-impact-resistance-human-health-health-canada-2002.html (accessed June, 2024).

^2^www.Canada.ca/en/public-health/services/surveillance/canadian-integrated-program-antimicrobial-resistance-surveillance-cipars/cipars-reports/2016-annual-report-summary.html (accessed March, 2024).

^3^www.Canada.ca/en/public-health/services/antibiotic-antimicrobial-resistance/antimicrobial-resistance-use-Canada-federal-framework-action.html (accessed February, 2024).

^4^https://www.cahi-icsa.ca/policy-and-outreach/antimicrobial-stewardship (accessed March, 2024).

^5^Antimicrobial Use Guidelines (Davis, CA: ISCAID). Available online at: www.iscaid.org/ (accessed May, 2024).

^6^www.hhs.gov/sites/default/files/carb-national-action-plan-2020-2025.pdf (accessed May, 2024).

^7^ www.fda.gov/animalveterinary/development-approval-process/veterinary-feed-directive-vfd

^8^www.albertaanimalhealthsource.ca/content/cephalosporin-order-prohibition-goes-effect (accessed March, 2024).

^9^www.cdc.gov/narms/index.html (accessed June, 2024).

## References

[B1] Aarestrup F. M. Jensen V. F. Emborg H. D. Jacobsen E. Wegener H. C. (2010). Changes in the use of antimicrobials and the effects on productivity of swine farms in denmark. Am. J. Vet. Res. 71:726–733. 10.2460/ajvr.71.7.726 20594073

[B2] Abdelfattah E. M. Ekong P. S. Okello E. Chamchoy T. Karle B. M. Black R. A. . (2021). Epidemiology of antimicrobial resistance (AMR) on california dairies: descriptive and cluster analyses of AMR phenotype of fecal commensal bacteria isolated from adult cows. PeerJ 9:11108. 10.7717/peerj.11108 33976962 PMC8063881

[B3] Abdi R. Gillespie B. E. Vaughn J. Merrill C. Headrick S. I Ensermu D. B. . (2018). Antimicrobial resistance of *Staphylococcus aureus* isolates from dairy cows and genetic diversity of resistant isolates. Foodborne Pathog. Dis. 15, 449–458. 10.1089/fpd.2017.2362 29394099

[B4] Adator E. Narvaez-Bravo C. Zaheer R. Cook S. Tymensen L. Hannon S. . (2022). A one health comparative assessment of antimicrobial resistance in generic and extended-spectrum cephalosporin-resistant Escherichia coli from beef production, sewage and clinical settings. Microorganisms 8:885. 10.3390/microorganisms8060885 32545206 PMC7355928

[B5] Aguilar-Montes de Oca S. Talavera-Rojas M. Soriano-Vargas E. Barba-León J. Vazquez-Navarrete J. (2015). Determination of extended spectrum beta-lactamases/ampc beta-lactamases and plasmid-mediated quinolone resistance in Escherichia coli isolates obtained from bovine carcasses in Mexico. Trop. Anim. Health Prod. 47, 975–981. 10.1007/s11250-015-0818-3 25894820

[B6] Aguilar-Montes de Oca S. Talavera-Rojas M. Soriano-Vargas E. Barba-León J. Vázquez-Navarrete J. Acosta-Dibarrat J. . (2018). Phenotypic and genotypic profile of clinical and animal multidrug-resistant salmonella enterica isolates from mexico. J. Appl. Microbiol. 124, 67–74. 10.1111/jam.13615 29044980

[B7] Agunos A. Gow S. P. Léger D. F. Carson C. A. Deckert A. E. Bosman A. L. . (2019). Antimicrobial use and antimicrobial resistance indicators-integration of farm-level surveillance data from broiler chickens and turkeys in british columbia, Canada. Front Vet Sci 6:131. 10.3389/fvets.2019.00131 31131285 PMC6509235

[B8] almada Corral A. Cordero-Ortiz M. LD B.-H. Calderén-Montoya S. Bolado-Martínez E. Sánchez-Mariñez R. . (2023). Evaluation of antimicrobial susceptibility and genetic profiles (ERIC-PCR) of enterococcus species isolated from chicken viscera. Biotecnia 25:169–175. 10.18633/biotecnia.v25i1.1869

[B9] Anomaly J. (2015). What's wrong with factory farming? Public Health Ethics 8:246–254. 10.1093/phe/phu001 36540869 PMC9757169

[B10] Antimicrobial Stewardship in Companion Animal Practice (2015). Antimicrobial stewardship in companion animal practice. J. Am. Vet. Med. Assoc. 246, 287–288. 10.2460/javma.246.3.287 25587725

[B11] Antimicrobial Use and Stewardship (Aus) Program Report to the Legislature, California, USA (2019). Available online at: https://www.cdfa.ca.gov/ahfss/aus/docs/CDFA_AUS_Report_2019.pdf

[B12] Aradanas M. Poljak Z. Fittipaldi N. Ricker N. Farzan A. (2021). Serotypes, virulence-associated factors, and antimicrobial resistance of streptococcus suis isolates recovered from sick and healthy pigs determined by whole-genome sequencing. Front Vet Sci 8:742345. 10.3389/fvets.2021.742345 34796225 PMC8593187

[B13] Arndt E. R. Farzan A. MacInnes J. I. Friendship R. M. (2019). Antimicrobial resistance of streptococcus suis isolates recovered from clinically ill nursery pigs and from healthy pigs at different stages of production. Can. Vet. J. 60, 519–522. 31080266 PMC6463947

[B14] Asaaga F. Young J. C. Srinivas P. N. Seshadri T. Oommen M. A. Rahman M. . (2022). Co-production of knowledge as part of a onehealth approach to better control zoonotic diseases. PLOS Glob. Public Health 2:e0000075. 10.1371/journal.pgph.0000075 36962247 PMC10021618

[B15] Asaduzzaman M. Rousham E. Unicomb L. . (2022). Spatiotemporal distribution of antimicrobial resistant organisms in different water environments in urban and rural settings of Bangladesh. Sci. Total Environ. 831:154890. 10.1016/j.scitotenv.2022.154890 35364179

[B16] Awosile B. McClure J. Sanchez J. C R.-L. J. Keefe G. L. C. . (2018). *Salmonella enterica* and extended-spectrum cephalosporin-resistant Escherichia coli recovered from holstein dairy calves from 8 farms in new Brunswick, Canada. J. Dairy Sci. 101, 3271–3284. 10.3168/jds.2017-13277 29428755

[B17] Awosile B. McClure J. Sanchez J. VanLeeuwen J. Rodriguez-Lecompte J. Keefe G. . (2017). Short communication: extended-spectrum cephalosporin-resistant *Escherichia coli* in colostrum from New Brunswick, Canada, dairy cows harbor blacmy-2 and blatem resistance genes. J. Dairy Sci. 100, 7901–7905. 10.3168/jds.2017-12941 28780105

[B18] Beck K. M. Waisglass S. E. Dick H. L. Weese J. S. (2012). Prevalence of meticillin-resistant *Staphylococcus pseudintermedius* (MRSP) from skin and carriage sites of dogs after treatment of their meticillin-resistant or meticillin-sensitive staphylococcal pyoderma. Vet. Dermatol. 23, 369–e67. 10.1111/j.1365-3164.2012.01035.x 22364707

[B19] Beier R. Andrews K. Hume M. Sohail M. Harvey R. Poole T. . (2021). Disinfectant and antimicrobial susceptibility studies of *Staphylococcus aureus* strains and st398-MRSA and st5-mrsa strains from swine mandibular lymph node tissue, commercial pork sausage meat and swine feces. Microorganisms 9:2401. 10.3390/microorganisms9112401 34835526 PMC8621428

[B20] Boc A. Makarenkov V. (2011). Towards an accurate identification of mosaic genes and partial horizontal gene transfers. Nucleic Acids Res. 39, e144–e144. 10.1093/nar/gkr735 21917854 PMC3241670

[B21] Borgen K. Simonsen G. S. Sundsfjord A. Wasteson Y. Olsvik O. Kruse H. (2000). Continuing high prevalence of vana-type vancomycin-resistant enterococci on norwegian poultry farms three years after avoparcin was banned. J. Appl. Microbiol. 89, 478–485. 10.1046/j.1365-2672.2000.01137.x 11021580

[B22] Brown E. E. F. Cooper A. Carrillo C. Blais B. (2019). Selection of multidrug-resistant bacteria in medicated animal feeds. Front. Microbiol. 10:456. 10.3389/fmicb.2019.00456 30894847 PMC6414793

[B23] Cameron A. Zaheer R. Adator E. H. Barbieri R. Reuter T. McAllister T. A. (2019). Bacteriocin occurrence and activity in *Escherichia coli* isolated from bovines and wastewater. Toxins 11:475. 10.3390/toxins11080475 31443193 PMC6723558

[B24] Canizalez-Roman A. Velazquez-Roman J. Valdez-Flores M. A. Flores-Villaseñor H. Vidal J. E. Muro-Amador S. . (2019). Detection of antimicrobial-resistance diarrheagenic *Escherichia coli* strains in surface water used to irrigate food products in the northwest of mexico. Int. J. Food Microbiol. 304, 1–10. 10.1016/j.ijfoodmicro.2019.05.017 31146052

[B25] Carroll L. M. Wiedmann M. den Bakker H. Siler J. Warchocki S. Kent D. . (2017). Whole-genome sequencing of drug-resistant salmonella enterica isolates from dairy cattle and humans in new york and washington states reveals source and geographic associations. Appl. Environ. Microbiol. 83, e00140–e00117. 10.1128/AEM.00140-17 28389536 PMC5452826

[B26] Chriki S. Hocquette J. F. (2020). The myth of cultured meat: a review. Front. Nutr. 7:7. 10.3389/fnut.2020.00007 32118026 PMC7020248

[B27] Christidis T. Hurst M. Rudnick W. Pintar K. D. M. (2020). A comparative exposure assessment of foodborne, animal contact and waterborne transmission routes of salmonella in canada. Food Contr. 109:106899. 10.1016/j.foodcont.2019.106899

[B28] Cobo-Angel C. Gohar B. L. S. (2022). Values and risk perception shape canadian dairy farmers' attitudes toward prudent use of antimicrobials. Antibiotics 11:550. 10.3390/antibiotics11050550 35625194 PMC9137716

[B29] College of Veterinarians of Ontario (2017). Setting an Action Agenda for Veterinary Stewardship of Antibiotic Use in Food-Producing Animals in Ontario. Available online at: viewer.joomag.com/growing-forward-2-final-report-project-ii/0379250001512484479?short (accessed March, 2024).

[B30] Cormier A. C. Chalmers G. Cook S. R. Zaheer R. Hannon S. J. Booker C. W. . (2020). Presence and diversity of extended-spectrum cephalosporin resistance among *Escherichia coli* from urban wastewater and feedlot cattle in Alberta, Canada. Microb. Drug Resist. 26:300–309. 10.1089/mdr.2019.0112 31553261

[B31] Cox G. W. Parmley E. J. Avery B. P. Irwin R. J. Reid-Smith R. J. Deckert A. E. . (2021). A one-health genomic investigation of gentamicin resistance in salmonella from human and chicken sources in Canada, 2014 to 2017. Antimicrob. Agents Chemother. 65:e0096621. 10.1128/aac.00677-22 34570642 PMC8597779

[B32] Cunningham M. Azcarate-Peril M. A. Barnard A. Benoit V. andGrimaldi R. Guyonnet D. . (2021). Shaping the future of probiotics and prebiotics. Trends Microbiol. 29, 667–685. 10.1016/j.tim.2021.01.003 33551269

[B33] Dalton K. R. Rock C. Carroll K. C. Davis M. F. (2020). One health in hospitals: how understanding the dynamics of people, animals, and the hospital built-environment can be used to better inform interventions for antimicrobial-resistant gram-positive infections. Antimicrob. Resist. Infect. Control 9:78. 10.1186/s13756-020-00737-2 32487220 PMC7268532

[B34] Dargatz D. Kopral C. Erdman M. Fedorka-Cray P. (2016). Prevalence and antimicrobial resistance of salmonella isolated from cattle feces in united states feedlots in 2011. Foodborne Pathog. Dis. 13, 483–489. 10.1089/fpd.2016.2128 27464334

[B35] Davedow T. Narvaez-Bravo C. Zaheer R. Sanderson H. A R.-G. Klima C. . (2020). Investigation of a reduction in tylosin on the prevalence of liver abscesses and antimicrobial resistance in enterococci in feedlot cattle. Front Vet Sci 7:3389. 10.3389/fvets.2020.00090 32185186 PMC7059211

[B36] Delgado-Suárez E. J. Ortíz-López R. Gebreyes W. A. Allard M. W. Barona-Gómez F. Rubio-Lozano M. S. (2019). Genomic surveillance links livestock production with the emergence and spread of multi-drug resistant non-typhoidal salmonella in Mexico. J. Microbiol. 57:271–280. 10.1007/s12275-019-8421-3 30721457

[B37] Delgado-Suárez E. J. Palós-Guitérrez T. Ruíz-López F. A. Hernández Pérez C. F. Ballesteros-Nova N. E. Soberanis-Ramos O. . (2021). Genomic surveillance of antimicrobial resistance shows cattle and poultry are a moderate source of multi-drug resistant non-typhoidal salmonella in mexico. PLoS ONE 16:e0243681. 10.1371/journal.pone.0243681 33951039 PMC8099073

[B38] Dewey C. E. (1999). Use of antimicrobials in swine feeds in the united states. Swine Health Prod 7, 19–25.

[B39] Dharmarha V. Guron G. Boyer R. R. Niemira B. A. Pruden A. Strawn L. K. . (2019). Gamma irradiation influences the survival and regrowth of antibiotic-resistant bacteria and antibiotic-resistance genes on romaine lettuce. Front. Microbiol. 10:710. 10.3389/fmicb.2019.00710 31024491 PMC6465624

[B40] Dierikx C. M. van Duijkeren E. Schoormans A. H. van Essen-Zandbergen A. Veldman K. Kant A. . (2012). Occurrence and characteristics of extended-spectrum-beta-lactamase and ampc-producing clinical isolates derived from companion animals and horses. J. Antimicrob. Chemother. 67, 1368–1374. 10.1093/jac/dks049 22382469

[B41] Doster E. Pinnell L. Noyes N. Parker J. Anderso C. Booker C. . (2022). Evaluating the effects of antimicrobial drug use on the ecology of antimicrobial resistance and microbial community structure in beef feedlot cattle. Front. Microbiol. 13:970358. 10.3389/fmicb.2022.970358 36583056 PMC9792868

[B42] Dramé O. Leclair D. Parmley E. J. Deckert A. Ouattara B. Daignault D. . (2020). Antimicrobial resistance of campylobacter in broiler chicken along the food chain in Canada. Foodborne Pathog. Dis. 17, 512–20. 10.1089/fpd.2019.2752 32130036 PMC7415884

[B43] Drouillard J. S. (2018). Current situation and future trends for beef production in the United States of America - a review. Asian-Australas. J. Anim. Sci. 31, 1007–1016. 10.5713/ajas.18.0428 29973030 PMC6039332

[B44] Dungan R. S. McKinney C. W. Leytem A. B. (2018). Tracking antibiotic resistance genes in soil irrigated with dairy wastewater. Sci. Total Environ. 635:1477–1483. 10.1016/j.scitotenv.2018.04.020 29710598

[B45] Dunlop R. H. McEwen S. A. Meek A. H. Friendship R. A. Clarke R. C. Black W. D. (1998). Antimicrobial drug use and related management practices among Ontario swine producers. Can. Vet. J. 39, 87–96. 10051955 PMC1539899

[B46] Dutil L. Irwin R. Finley R. N. L. K. Avery B. Boerlin P. Bourgault A. M. . (2010). Ceftiofur resistance in salmonella enterica serovar heidelberg from chicken meat and humans, Canada. Emerging Infect. Dis. 16, 48–54. 10.3201/eid1601.090729 20031042 PMC2874360

[B47] Ekakoro J. E. Caldwell M. Strand E. B. Okafor C. C. (2019). Drivers, alternatives, knowledge, and perceptions towards antimicrobial use among tennessee beef cattle producers: a qualitative study. BMC Vet. Res. 15:16. 10.1186/s12917-018-1731-6 30616648 PMC6323766

[B48] Ekhlas D. Argüello H. Leonard F. C. Manzanilla E. G. Burgess C. M. (2023). Insights on the effects of antimicrobial and heavy metal usage on the antimicrobial resistance profiles of pigs based on culture-independent studies. Vet. Res. 54:14. 10.1186/s13567-023-01143-3 36823539 PMC9951463

[B49] Enciso-Martínez Y. Barrios-Villa E. Sepúlveda-Moreno C. O. Ballesteros-Monrreal M. G. Valencia-Rivera D. E. González-Aguilar G. A. . (2022). Prevalence of antibiotic-resistant *E. coli* strains in a local farm and packing facilities of honeydew melon in Hermosillo, Sonora, Mexico. Antibiotics 11:1789. 10.3390/antibiotics11121789 36551446 PMC9774811

[B50] Engberg J. Aarestrup F. M. Taylor D. E. Gerner-Smidt P. Nachamkin I. (2001). Quinolone and macrolide resistance in campylobacter jejuni and c. coli: resistance mechanisms and trends in human isolates. Emerging Infect. Dis. 7:491. 10.3201/eid0701.010104 11266291 PMC2631682

[B51] Fonseca M. Heider L. Stryhn H. McClure J. Léger D. Rizzo D. . (2023). Frequency of isolation and phenotypic antimicrobial resistance of fecal salmonella enterica recovered from dairy cattle in Canada. J. Dairy Sci. 23, 00760–00769. 10.3168/jds.2023-23937 37863297

[B52] Fujita A. W. Werner K. Jacob J. T. Tschopp R. Mamo G. Mihret A. . (2022). Antimicrobial resistance through the lens of one health in ethiopia: a review of the literature among humans, animals, and the environment. Int. J. Infect. Dis. 119, 120–129. 10.1016/j.ijid.2022.03.041 35358724 PMC9107604

[B53] Galiot L. Monger X. C. Vincent A. T. (2023). Studying the association between antibiotic resistance genes and insertion sequences in metagenomes: challenges and pitfalls. Antibiotics 12:175. 10.3390/antibiotics12010175 36671375 PMC9854595

[B54] Gao F. Z. He L. Y. Bai H. He L. X. Zhang M. Chen Z. Y. . (2023). Airborne bacterial community and antibiotic resistome in the swine farming environment: metagenomic insights into livestock relevance, pathogen hosts and public risks. Environ. Int. 172:107751. 10.1016/j.envint.2023.107751 36680804

[B55] Ghosh C. Sarkar P. Issa R. Haldar J. (2019). Alternatives to conventional antibiotics in the era of antimicrobial resistance. Trends Microbiol. 27, 323–338. 10.1016/j.tim.2018.12.010 30683453

[B56] Godínez-Oviedo A. Sampedro F. Bowman J. P. andGarcés-Vega F. J. Hernández-Iturriaga M. (2023). Risk ranking of food categories associated with salmonella enterica contamination in the central region of mexico. Risk Anal. 43, 308–323. 10.1111/risa.13907 35383989

[B57] Gou M. Hu H. W. Zhang Y. J. Wang J. T. Hayden H. Tang Y. Q. . (2018). Aerobic composting reduces antibiotic resistance genes in cattle manure and the resistome dissemination in agricultural soils. Sci. Total Environ. 612, 1300–1310. 10.1016/j.scitotenv.2017.09.028 28898936

[B58] Graham J. P. Evans S. L. Price L. B. Silbergeld E. K. (2009). Fate of antimicrobial-resistant enterococci and staphylococci and resistance determinants in stored poultry litter. Environ. Res. 109, 682–689. 10.1016/j.envres.2009.05.005 19541298

[B59] Graham J. P. Nachman K. E. (2010). Managing waste from confined animal feeding operations in the United States: the need for sanitary reform. J. Water Health 8, 646–670. 10.2166/wh.2010.075 20705978

[B60] Guzmán-Rodríguez J. Salinas-Pérez E. León-Galván F. Barboza-Corona J. E. Valencia-Posadas M. Ávila-Ramos F. . (2021). Relationship between antibiotic resistance and biofilm production of *Staphylococcus aureus* isolates from bovine mastitis. Rev. Mex. De cienc. Pecuarias 12, 1117–1132. 10.22319/rmcp.v12i4.5645

[B61] Haenni M. Saras E. Châtre P. Médaille C. Bes M. Madec J. Y. . (2012). A usa300 variant and other human-related methicillin-resistant *Staphylococcus aureus* strains infecting cats and dogs in france. J. Antimicrob. Chemother. 67:326–329. 10.1093/jac/dkr499 22146878

[B62] Hailu W. Helmy Y. A. Carney-Knisely G. Kauffman M. Fraga D. Rajashekara G. (2021). Prevalence and antimicrobial resistance profiles of foodborne pathogens isolated from dairy cattle and poultry manure amended farms in northeastern Ohio, the United States. Antibiotics 10:1450. 10.3390/antibiotics10121450 34943663 PMC8698512

[B63] Hau S. J. Frana T. Sun J. Davies P. R. Nicholson T. L. (2017). Zinc resistance within swine-associated methicillin-resistant staphylococcus aureus isolates in the united states is associated with multilocus sequence type lineage. Appl Environ Microbiol 83:e00756-17. 10.1128/AEM.00756-17 28526788 PMC5514667

[B64] Hau S. J. Haan J. S. Davies P. R. Frana T. Nicholson T. L. (2018). Antimicrobial resistance distribution differs among methicillin resistant *staphylococcus aureus* sequence type (ST) 5 isolates from health care and agricultural sources. Front. Microbiol. 9:2102. 10.3389/fmicb.2018.02102 30258418 PMC6143795

[B65] Hayer S. Rovira A. Olsen K. Johnson T. Vannucci F. Rendahl A. . (2020a). Prevalence and trend analysis of antimicrobial resistance in clinical *Escherichia coli* isolates collected from diseased pigs in the usa between 2006 and 2016. Transbound. Emerg. Dis. 67, 1930–1941. 10.1111/tbed.13528 32097517

[B66] Hayer S. S. Lim S. Hong S. Elnekave E. Johnson T. Rovira A. . (2020b). Genetic determinants of resistance to extended-spectrum cephalosporin and fluoroquinolone in *Escherichia coli* isolated from diseased pigs in the united states. mSphere 5, e00990–e00920. 10.1128/mSphere.00990-20 33115839 PMC8534314

[B67] Heiman K. E. Mody R. K. Johnson S. D. Griffin P. M. Gould L. H. (2015). Escherichia coli o157 outbreaks in the United States, 2003-2012. Emerg. Infect. Dis. 21, 1293–1301. 10.3201/eid2108.141364 26197993 PMC4517704

[B68] Holman D. B. Hao X. Topp E. Yang H. E. Alexander T. W. (2016). Effect of co-composting cattle manure with construction and demolition waste on the archaeal, bacterial, and fungal microbiota, and on antimicrobial resistance determinants. PLoS ONE 11:e0157539. 10.1371/journal.pone.0157539 27300323 PMC4907429

[B69] Holmer I. Salomonsen C. M. Jorsal S. E. Astrup L. B. Jensen V. F. Borck Høg B. . (2019). Antibiotic resistance in porcine pathogenic bacteria and relation to antibiotic usage. BMC Vet. Res. 15:449. 10.1186/s12917-019-2162-8 31829171 PMC6907208

[B70] Holmes A. H. Moore L. S. Sundsfjord A. Steinbakk M. Regmi S. Karkey A. . (2016). Understanding the mechanisms and drivers of antimicrobial resistance. Lancet 387, 176–187. 10.1016/S0140-6736(15)00473-0 26603922

[B71] Hsieh Y. C. Poole T. L. Runyon M. Hume M. and J. H. T. (2016). Prevalence of nontyphoidal salmonella and salmonella strains with conjugative antimicrobial-resistant serovars contaminating animal feed in texas. J. Food Prot. 79, 194–204. 10.4315/0362-028X.JFP-15-163 26818979

[B72] Huang H. Brooks B. W. Lowman R. Carrillo C. D. (2015). Campylobacter species in animal, food, and environmental sources, and relevant testing programs in Canada. Can. J. Microbiol. 61, 701–721. 10.1139/cjm-2014-0770 26422448

[B73] Huber L. Agunos A. Gow S. P. Carson C. A. Van Boeckel T. P. (2021). Reduction in antimicrobial use and resistance to salmonella, campylobacter, and *Escherichia coli* in broiler chickens, Canada, 2013-2019. Emerging Infect. Dis. 27, 2434–2444. 10.3201/eid2709.204395 34424161 PMC8386787

[B74] Huijbers P. M. Blaak H. de Jong M. C. Graat E. A. Vandenbroucke-Grauls C. M. de Roda Husman A. M. (2015). Role of the environment in the transmission of antimicrobial resistance to humans: a review. Environm. Sci. Technol. 49, 11993–12004. 10.1021/acs.est.5b02566 26355462

[B75] Hull D. M. Harrell E. van Vliet A. H. M. Correa M. Thakur S. (2021). Antimicrobial resistance and interspecies gene transfer in campylobacter coli and campylobacter jejuni isolated from food animals, poultry processing, and retail meat in north carolina 2018-2019. PLoS ONE 16:e0246571. 10.1371/journal.pone.0246571 33571292 PMC7877606

[B76] Hume M. E. (2011). Historic perspective: prebiotics, probiotics, and other alternatives to antibiotics. Poult. Sci. 90, 2663–2669. 10.3382/ps.2010-01030 22010256

[B77] Ibekwe A. M. Bhattacharjee A. S. Phan D. Ashworth D. Schmidt M. P. Murinda S. E. . (2023). Potential reservoirs of antimicrobial resistance in livestock waste and treated wastewater that can be disseminated to agricultural land. Sci. Total Environ. 872:162194. 10.1016/j.scitotenv.2023.162194 36781130

[B78] Ida J. A. Wilson W. M. Nydam D. V. Gerlach S. C. Kastelic J. P. Russell E. R. . (2023). Contextualized understandings of dairy farmers' perspectives on antimicrobial use and regulation in Alberta, Canada. J. Dairy Sci. 106, 547–564. 10.3168/jds.2021-21521 36424321 PMC10957287

[B79] Igwaran A. Okoh A. (2019). Human campylobacteriosis: a public health concern of global importance. Heliyon 5:e02814. 10.1016/j.heliyon.2019.e02814 31763476 PMC6861584

[B80] Innes G. K. Randad P. R. Korinek A. Davis M. F. Price L. B. So A. D. . (2020). External societal costs of antimicrobial resistance in humans attributable to antimicrobial use in livestock. Annu. Rev. Public Health 41, 141–157. 10.1146/annurev-publhealth-040218-043954 31910712 PMC7199423

[B81] Jahanbakhsh S. Kabore K. Fravalo P. Letellier A. Fairbrother J. (2015). Impact of medicated feed along with clay mineral supplementation on *Escherichia coli* resistance to antimicrobial agents in pigs after weaning in field conditions. Res. Vet. Sci. 102, 72–79. 10.1016/j.rvsc.2015.07.014 26412523

[B82] Jahanbakhsh S. Letellier A. Fairbrother J. (2016a). Circulating of cmy-2 β-lactamase gene in weaned pigs and their environment in a commercial farm and the effect of feed supplementation with a clay mineral. J. Appl. Microbiol. 121, 136–148. 10.1111/jam.13166 27138244

[B83] Jahanbakhsh S. Smith M. Kohan-Ghadr H. Letellier A. Abraham S. Trott D. . (2016b). Dynamics of extended-spectrum cephalosporin resistance in pathogenic *Escherichia coli* isolated from diseased pigs in Quebec, Canada. Int. J. Antimicrob. Agents 48, 194–202. 10.1016/j.ijantimicag.2016.05.001 27286922

[B84] JAwosile B. Heider L. Saab M. McClure J. (2018). Antimicrobial resistance in mastitis, respiratory and enteric bacteria isolated from ruminant animals from the atlantic provinces of Canada from 1994-2013. Can. Vet. J. 59, 1099–1104. 30510316 PMC6135275

[B85] Jeamsripong S. Li X. Aly S. S. Su Z. Pereira R. V. Atwill E. R. (2021). Antibiotic resistance genes and associated phenotypes in *Escherichia coli* and enterococcus from cattle at different production stages on a dairy farm in central california. Antibiotics 10:1042. 10.3390/antibiotics10091042 34572624 PMC8471271

[B86] Kahn L. H. (2017). Antimicrobial resistance: a one health perspective. Trans. R. Soc. Trop. Med. Hyg. 111, 255–260. 10.1093/trstmh/trx050 29044373

[B87] Kaur K. Singh S. Kaur R. (2024). Impact of antibiotic usage in food-producing animals on food safety and possible antibiotic alternatives. The Microbe 4:100097. 10.1016/j.microb.2024.100097

[B88] Khanna T. Friendship R. Dewey C. Weese J. S. (2008). Methicillin resistant *Staphylococcus aureus* colonization in pigs and pig farmers. Vet. Microbiol. 128, 298–303. 10.1016/j.vetmic.2007.10.006 18023542

[B89] Kimera Z. I. Mshana S. E. Rweyemamu M. M. Mboera L. E. Matee M. I. (2020). Antimicrobial use and resistance in food-producing animals and the environment: an african perspective. Antimicrob. Resist. Infect. Cont. 9, 1–12. 10.1186/s13756-020-0697-x 32122406 PMC7053060

[B90] Kotwani A. Joshi J. Kaloni D. (2021). Pharmaceutical effluent: a critical link in the interconnected ecosystem promoting antimicrobial resistance. Environ. Sci. Pollut. Res. Int. 28:32111–32124. 10.1007/s11356-021-14178-w 33929671 PMC8086231

[B91] Lee K. Atwill E. Pitesky M. Huang A. Lavelle K. Rickard M. . (2018). Antimicrobial resistance profiles of non-typhoidal salmonella from retail meat products in california, 2018. Front. Microbiol. 16:835699. 10.3389/fmicb.2022.835699 35369434 PMC8966841

[B92] Lees P. Pelligand L. Giraud E. Toutain P. (2021). A history of antimicrobial drugs in animals: Evolution and revolution. J. Vet. Pharmacol. Ther. 44, 137–171. 10.1111/jvp.12895 32725687

[B93] Léger D. F. Anderson M. E. C. Béedard F. D. Burns T. Carson C. A. Deckert A. E. . (2022). Canadian collaboration to identify a minimum dataset for antimicrobial use surveillance for policy and intervention development across food animal sectors. Antibiotics 11:226. 10.3390/antibiotics11020226 35203828 PMC8868246

[B94] Levent G. Schlochtermeier A. Vinasco J. Jennings J. J R. Ives S. . (2022). Long-term effects of single-dose cephalosporin or macrolide use on the prevalence of AMPC and extended-spectrum β-lactamase producing *Escherichia coli* in the feces of beef cattle. Microorganisms 10:2071. 10.3390/microorganisms10102071 36296347 PMC9610231

[B95] Lhermie G. Tauer L. W. Gröhn Y. T. (2018). The farm cost of decreasing antimicrobial use in dairy production. PLoS ONE 13:e0194832. 10.1371/journal.pone.0194832 29566103 PMC5864045

[B96] Liljebjelke K. A. Hofacre C. L. White D. G. Ayer S. Lee M. D. Maurer J. J. (2017). Diversity of antimicrobial resistance phenotypes in salmonella isolated from commercial poultry farms. Front. Vet. Sci. 4:96. 10.3389/fvets.2017.00096 28691011 PMC5482141

[B97] Lone A. Mottawea W. Mehdi Y. andHammami R. (2022). Bacteriocinogenic probiotics as an integrated alternative to antibiotics in chicken production - why and how? Crit. Rev. Food Sci. Nutr. 62, 8744–8760. 10.1080/10408398.2021.1932722 34060404

[B98] Lopatkin A. J. Meredith H. R. Srimani J. K. Pfeiffer C. Durrett R. You L. (2017). Persistence and reversal of plasmid-mediated antibiotic resistance. Nat. Commun. 8:1689. 10.1038/s41467-017-01532-1 29162798 PMC5698434

[B99] Majumder S. Jung D. Ronholm J. George S. (2021). Prevalence and mechanisms of antibiotic resistance in *Escherichia coli* isolated from mastitic dairy cattle in Canada. BMC Microbiol. 21:222. 10.1186/s12866-021-02280-5 34332549 PMC8325273

[B100] Makarenkov V. Mazoure B. Rabusseau G. Legendre P. (2021). Horizontal gene transfer and recombination analysis of SARS-CoV-2 genes helps discover its close relatives and shed light on its origin. BMC Ecol. Evolut. 21:1–18. 10.1186/s12862-020-01732-2 33514319 PMC7817968

[B101] Mandujano A. Cortés-Espinosa D. Vásquez-Villanueva J. Guel P. Rivera G. Juárez-Rendón K. . (2023). Extended-spectrum β-lactamase-producing *Escherichia coli* isolated from food-producing animals in Tamaulipas, Mexico. Antibiotics 12:1010. 10.3390/antibiotics12061010 37370329 PMC10294937

[B102] Manyi-Loh C. Mamphweli S. Meyer E. Okoh A. (2018). Antibiotic use in agriculture and its consequential resistance in environmental sources: potential public health implications. Molecules 23:795. 10.3390/molecules23040795 29601469 PMC6017557

[B103] Maradiaga M. Echeverry A. Miller M. den Bakker H. C. Nightingale K. Cook P. . (2019). Characterization of antimicrobial resistant (AMR) salmonella enterica isolates associated with cattle at harvest in mexico. Meat and Muscle Biol. 3:53. 10.22175/mmb2017.10.0053

[B104] Martak D. Henriot C. Hocquet D. (2024). Environment, animals, and food as reservoirs of antibiotic-resistant bacteria for humans: one health or more? Infect Dis Now 54:104895. 10.1016/j.idnow.2024.104895 38548016

[B105] Martínez-Vázquez A. Guardiola-Avila I. Flores-Magallón R. (2021). Detection of multi-drug resistance and methicillin-resistant *Staphylococcus aureus* (MRSA) isolates from retail meat in tamaulipas, mexico. Ann. Microbiol. 71:16. 10.1186/s13213-021-01627-7

[B106] Martínez-Vázquez A. Rivera-Sanchez G. Lira-Mendez K. Reyes-Lopez M.A. Bocanegra-Garcia V. (2018). Prevalence, antimicrobial resistance and virulence genes of escherichia coli isolated from retail meat in tamaulipas, mexico. J Glob Antimicrob Resist 14, 266–272. 10.1016/j.jgar.2018.02.016 29501529

[B107] Massé J. Lardé H. Fairbrother J. M. Roy J. P. Francoz D. Dufour S. . (2021). Prevalence of antimicrobial resistance and characteristics of Escherichia coli isolates from fecal and manure pit samples on dairy farms in the province of Quebec, Canada. Front. Vet. Sci. 8:654125. 10.3389/fvets.2021.654125 34095273 PMC8175654

[B108] Massé J. Vanier G. Fairbrother J. de Lagarde M. Arsenault J. Francoz D. . (2023). Description of antimicrobial-resistant Escherichia coli and their dissemination mechanisms on dairy farms. Vet. Sci. 10:242. 10.3390/vetsci10040242 37104397 PMC10144642

[B109] Mays C. Garza G. L. Waite-Cusic J. Radniecki T. S. Navab-Daneshmand T. (2021). Impact of biosolids amendment and wastewater effluent irrigation on enteric antibiotic-resistant bacteria - a greenhouse study. Water Res. X 13:100119. 10.1016/j.wroa.2021.100119 34585133 PMC8452883

[B110] McCubbin K. D. Anholt R. M. de Jong E. Ida J. A. Nóbrega D. B. Kastelic J. P. . (2021). Knowledge gaps in the understanding of antimicrobial resistance in Canada. Front Public Health 9:726484. 10.3389/fpubh.2021.726484 34778169 PMC8582488

[B111] Mehrotra M. Li X. Z. Ireland M. (2017). Enhancing antimicrobial stewardship by strengthening the veterinary drug regulatory framework. Can. Commun. Dis. Rep. 43:220–223. 10.14745/ccdr.v43i11a02 29770050 PMC5764736

[B112] Mohsin M. Farooq U. Hartmann M. Brogden S. Kreienbrock L. Stoffregen J. (2023). Case study: using a shared international database to document veterinary consumption of antibiotics in Pakistan. Antibiotics 12:394. 10.3390/antibiotics12020394 36830304 PMC9952550

[B113] Mollenkopf D. F. Stull J. W. Mathys D. A. Bowman A. S. Feicht S. M. Grooters S. V. . (2017). Carbapenemase-producing enterobacteriaceae recovered from the environment of a swine farrow-to-finish operation in the united states. Antimicrob. Agents Chemother. 61, e01298–e01216. 10.1128/AAC.01298-16 27919894 PMC5278694

[B114] Montoro-Dasi L. Villagra A. Sevilla-Navarro S. Pérez-Gracia M. Vega S. Marin C. (2020). The dynamic of antibiotic resistance in commensal Escherichia coli throughout the growing period in broiler chickens: fast-growing vs. slow-growing breeds. Poult Sci. 10, 1591–1597. 10.1016/j.psj.2019.10.080 32111325 PMC7587802

[B115] Mora-Hernández Y. Vera Murguía E. Stinenbosch J. Hernández Jauregui P. van Dijl J. M. Buist G. (2021). Molecular typing and antimicrobial resistance profiling of 33 mastitis-related *Staphylococcus aureus* isolates from cows in the comarca lagunera region of mexico. Sci. Rep. 11:6912. 10.1038/s41598-021-86453-2 33767356 PMC7994548

[B116] Moreno M. A. (2012). Survey of quantitative antimicrobial consumption in two different pig finishing systems. Vet. Rec. 171:325. 10.1136/vr.100818 22915683

[B117] Morris C. Wickramasingha D. Abdelfattah E. Pereira R. Okello E. Maier G. (2023). Prevalence of antimicrobial resistance in fecal *Escherichia coli* and enterococcus spp. isolates from beef cow-calf operations in northern california and associations with farm practices. Front. Microbiol. 14:1086203. 10.3389/fmicb.2023.1086203 36910206 PMC9996069

[B118] Mujahid S. Hansen M. Miranda R. Newsom-Stewart K. Rogers J. E. (2023). Prevalence and antibiotic resistance of salmonella and campylobacter isolates from raw chicken breasts in retail markets in the united states and comparison to data from the plant level. Life 13:642. 10.3390/life13030642 36983798 PMC10055585

[B119] Murray R. Tien Y. C. Scott A. Topp E. (2019). The impact of municipal sewage sludge stabilization processes on the abundance, field persistence, and transmission of antibiotic resistant bacteria and antibiotic resistance genes to vegetables at harvest. Sci. Total Environ. 651, 680–1687. 10.1016/j.scitotenv.2018.10.030 30316087

[B120] Narvaez-Bravo C. Taboada E. Mutschall S. and M. A. (2017). Epidemiology of antimicrobial resistant *Campylobacter spp*. isolated from retail meats in Canada. Int. J. Food Microbiol. 253, 43–47. 10.1016/j.ijfoodmicro.2017.04.019 28477522

[B121] Nathan C. (2020). Resisting antimicrobial resistance. Nat. Rev. Microbiol. 18:259–260. 10.1038/s41579-020-0348-5 32300248

[B122] Naushad S. Nobrega D. Naqvi S. Barkema H. De Buck J. (2020). Genomic analysis of bovine *Staphylococcus aureus* isolates from milk to elucidate diversity and determine the distributions of antimicrobial and virulence genes and their association with mastitis. mSystems 5, e00063–e00020. 10.1128/mSystems.00063-20 32636332 PMC7343304

[B123] Nicholson T. Bayles D. (2022). Comparative virulence and antimicrobial resistance distribution of *Streptococcus suis* isolates obtained from the united states. Front. Microbiol. 13:1043529. 10.3389/fmicb.2022.1043529 36439859 PMC9687383

[B124] Nobrega D. De Buck J. Barkema H. (2018a). Antimicrobial resistance in non-aureus staphylococci isolated from milk is associated with systemic but not intramammary administration of antimicrobials in dairy cattle. J. Dairy Sci. 101:7425–7436. 10.3168/jds.2018-14540 29729922

[B125] Nobrega D. Naushad S. Naqvi S. Condas L. Saini V. Kastelic J. P. (2018b). Prevalence and genetic basis of antimicrobial resistance in non-aureus staphylococci isolated from Canadian dairy herds. Front. Microbiol. 8:256. 10.3389/fmicb.2018.00256 29503642 PMC5820348

[B126] Nova Nayarit-Ballesteros M. María Salud Rubio-Lozano D. Enrique Delgado-Suárez M. Danilo Méndez-Medina D. Diego Braña-Varela D. Oscar Rodas-Suárez D. (2016). Perfil de resistencia a antibióticos de serotipos de salmonella spp. aislados de carne de res molida en la ciudad de méxico. Salud Pública De México 58, 371–377. 10.21149/spm.v58i3.7897 27598935

[B127] Novoa Rama E. Bailey M. Jones D. (2018). Prevalence, persistence, and antimicrobial resistance of campylobacter spp. from eggs and laying hens housed in five commercial housing systems. Foodborne Pathog. Dis. 15, 506–516. 10.1089/fpd.2017.2404 30124342

[B128] O'Neill J. (2016). Review on Antimicrobial Resistance: Tackling Drug-Resistant Infections Globally: Final Report and Recommendations. The Wellcome Trust and the UK Department of Health.

[B129] Otto S. Haworth-Brockman M. Miazga-Rodriguez M. Wierzbowski A. Saxinger L. (2022). Integrated surveillance of antimicrobial resistance and antimicrobial use: evaluation of the status in Canada (2014-2019). Can. Vet. J. 63, 161–170. 10.17269/s41997-021-00600-w 35110774 PMC8759327

[B130] Park J. Friendship R. M. Poljak Z. Weese J. S. Dewey C. E. (2013). An investigation of exudative epidermitis (greasy pig disease) and antimicrobial resistance patterns of staphylococcus hyicus and *Staphylococcus aureus* isolated from clinical cases. Can. Vet. J. 54, 139–144. 23904636 PMC3552588

[B131] Paulson J. A. Zaoutis T. E. Council on Environmental Health. (2015). Nontherapeutic use of antimicrobial agents in animal agriculture: Implications for pediatrics. Pediatrics 136, e1670–e1677. 10.1542/peds.2015-3630 26574594

[B132] Pinto C. E. Keestra S. M. Tandon P. Pickering A. J. Moodley A. Cumming O. . (2023). One health wash: an AMR-smart integrative approach to preventing and controlling infection in farming communities. BMJ Global Health 8:e011263. 10.1136/bmjgh-2022-011263 36882219 PMC10008318

[B133] Pires J. Huisman J. Bonhoeffer S. Van Boeckel T. (2021). Multidrug resistance dynamics in salmonella in food animals in the united states: an analysis of genomes from public databases. Microbiol. Spectr. 16:e0049521. 10.1128/Spectrum.00495-21 34704804 PMC8549754

[B134] Pornsukarom S. Thakur S. (2016). Assessing the impact of manure application in commercial swine farms on the transmission of antimicrobial resistant salmonella in the environment. PLoS ONE 11:e0164621. 10.1371/journal.pone.0164621 27755598 PMC5068702

[B135] Pourabedin M. Zhao X. (2015). Prebiotics and gut microbiota in chickens. FEMS Microbiol. Lett. 362:fnv122. 10.1093/femsle/fnv122 26208530

[B136] Prescott J. (2017). “History and current use of antimicrobial drugs in veterinary medicine,” in Antimicrobial Resistance in Bacteria from Livestock and Companion Animals, eds. F. Aarestrup, S. Schwarz, J. Shen, and L. Cavaco (Washington, D.C.: ASM Press).

[B137] Qian X. Gu J. Sun W. Wang X. J. Su J. Q. Stedfeld R. (2018). Diversity, abundance, and persistence of antibiotic resistance genes in various types of animal manure following industrial composting. J. Hazard. Mater 344:716–722. 10.1016/j.jhazmat.2017.11.020 29154097

[B138] Rahman M. Alam M. U. Luies S. K. Kamal A. Ferdous S. Lin A. . (2021). Contamination of fresh produce with antibiotic-resistant bacteria and associated risks to human health: a scoping review. Int. J. Environ. Res. Public Health 19:360. 10.3390/ijerph19010360 35010620 PMC8744955

[B139] Rama E. N. Bailey M. Kumar S. Leone C. Bakker H. C. d. . (2022). Prevalence and antimicrobial resistance of salmonella in conventional and no antibiotics ever broiler farms in the united states. Food Cont.135:108738. 10.1016/j.foodcont.2021.10873838331218

[B140] Randad P. Larsen J. Kaya H. Pisanic N. Ordak C. Price L. . (2021). Transmission of antimicrobial-resistant *Staphylococcus aureus* clonal complex 9 between pigs and humans, united states. Emerging Infect. Dis. 27, 740–748. 10.3201/eid2703.191775 33622471 PMC7920674

[B141] Rao S. Linke L. Magnuson R. Jauch L. Hyatt D. (2021). Antimicrobial resistance and interspecies gene transfer in *Campylobacter coli* and *Campylobacter jejuni* isolated from food animals, poultry processing, and retail meat in north carolina, 2018-2019. PLoS ONE 16:e0246571. 33571292 10.1371/journal.pone.0246571 PMC7877606

[B142] Redding L. E. Habing G. G. Tu V. Bittinger K. L. O'Day J. Pancholi P. . (2023). Infrequent intrahousehold transmission of *Clostridioides difficile* between pet owners and their pets. Zoonoses Public Health 10, 1–10. 10.1111/zph.13032 36779297 PMC10175142

[B143] Rhouma M. Tessier M. Aenishaenslin C. Sanders P. Carabin H. (2021). Should the increased awareness of the one health approach brought by the Covid-19 pandemic be used to further tackle the challenge of antimicrobial resistance? Antibiotics 10:464. 10.3390/antibiotics10040464 33923886 PMC8073751

[B144] Ribeiro L. F. Nespolo N. M. Rossi G. Fairbrother M. J. (2024). Exploring extended-spectrum beta-lactamase (ESBL)-producing *Escherichia coli* in food-producing animals and animal-derived foods. Pathogens 13:346. 10.3390/pathogens13040346 38668301 PMC11054374

[B145] Rima M. Rima M. Fajloun Z. Sabatier J. M. Bechinger B. Naas T. (2021). Antimicrobial peptides: a potent alternative to antibiotics. Antibiotics (Basel) 10:1095. 10.3390/antibiotics10091095 34572678 PMC8466391

[B146] Rodríguez-Medina N. Barrios-Camacho H. Duran-Bedolla J. Garza-Ramos U. (2019). *Klebsiella variicola*: an emerging pathogen in humans. Emerg. Microbes Infect. 8, 973–988. 10.1080/22221751.2019.1634981 31259664 PMC6609320

[B147] Roken M. Forfang K. Wasteson Y. Haaland A. H. Eiken H. G. Hagen S. B. . (2022). Antimicrobial resistance-do we share more than companionship with our dogs? J. Appl. Microbiol. 133, 1027–1039. 10.1111/jam.15629 35596927 PMC9542740

[B148] Romero Barrios P. Deckert A. Parmley E. J. Leclair D. (2020). Antimicrobial resistance profiles of *Escherichia coli* and salmonella isolates in Canadian broiler chickens and their products. Foodborne Pathog. Dis. 17, 672–678. 10.1089/fpd.2019.2776 32667209 PMC7692893

[B149] Rortveit G. Simonsen G. (2020). The primary care perspective on the Norwegian national strategy against antimicrobial resistance. Antibiotics 9:622. 10.3390/antibiotics9090622 32961691 PMC7558667

[B150] rudzynski K. S. C. (2015). Honey glycoproteins containing antimicrobial peptides, jelleins of the major royal jelly protein 1, are responsible for the cell wall lytic and bactericidal activities of honey. PLoS ONE 10:e0120238. 10.1371/journal.pone.0120238 25830314 PMC4382210

[B151] Salaheen S. Cao H. Sonnier J. Kim S. Del Collo L. Hovingh E. . (2019). Diversity of extended-spectrum cephalosporin-resistant *Escherichia coli* in feces from calves and cows on pennsylvania dairy farms. Foodborne Pathog. Dis. 16, 368–370. 10.1089/fpd.2018.2579 30715902

[B152] Salam M. A. Al-Amin M. Y. Salam M. T. Pawar J. S. Akhter N. Rabaan A. A. . (2023). Antimicrobial resistance: a growing serious threat for global public health. Healthcare 11:1946. 10.3390/healthcare11131946 37444780 PMC10340576

[B153] Salgado-Ruiz T. B. Rodríguez A. Gutiérrez D. Martínez B. García P. Espinoza-Ortega A. . (2015). Molecular characterization and antimicrobial susceptibility of *Staphylococcus aureus* from small-scale dairy systems in the highlands of Central México. Dairy Sci. Technol. 95, 181–196. 10.1007/s13594-014-0195-0

[B154] Sancheza H. M. Echeverria C. Thulsiraj V. Faust A. Z. Flores A. Laitz M. . (2016). Antibiotic resistance in airborne bacteria near conventional and organic beef cattle farms in California, USA. Water Air Soil Pollut. 227:280. 10.1007/s11270-016-2979-8

[B155] Sapkota A. R. Lefferts L. Y. McKenzie S. Walker P. (2007). What do we feed to food-production animals? A review of animal feed ingredients and their potential impacts on human health. Environ. Health Perspect. 115, 663–670. 10.1289/ehp.9760 17520050 PMC1867957

[B156] Schwarz S. Loeffler A. Kadlec K. (2017). Bacterial resistance to antimicrobial agents and its impact on veterinary and human medicine. Vet. Dermatol. 28, 82–e19. 10.1111/vde.12362 27581211

[B157] Scott A. Tien Y. C. Drury C. F. Reynolds W. D. Topp E. (2018). Enrichment of antibiotic resistance genes in soil receiving composts derived from swine manure, yard wastes, or food wastes, and evidence for multiyear persistence of swine *Clostridium spp*. Can. J. Microbiol. 64, 201–208. 10.1139/cjm-2017-0642 29342372

[B158] Shrestha R. Agunos A. Gow S. Deckert A. Varga C. (2022). Associations between antimicrobial resistance in fecal *Escherichia coli* isolates and antimicrobial use in Canadian turkey flocks. Front. Microbiol. 29:954123. 10.3389/fmicb.2022.954123 35966666 PMC9372513

[B159] Sibbald B. (2012). Farm-grown superbugs: while the world acts, Canada dawdles. CMAJ 184:1553. 10.1503/cmaj.120561 22664761 PMC3470614

[B160] Smith O. M. Snyder W. E. Owen J. P. (2020). Are we overestimating risk of enteric pathogen spillover from wild birds to humans? Biol. Rev. Camb. Philos. Soc. 95:95. 10.1111/brv.12581 32003106 PMC7317827

[B161] Sodagari H. Varga C. (2023). Evaluating antimicrobial resistance trends in commensal *Escherichia coli* isolated from cecal samples of swine at slaughter in the United States, 2013-2019. Microorganisms 11:1033. 10.3390/microorganisms11041033 37110456 PMC10142105

[B162] Sodagari H. Varga C. Habib I. Sahibzada S. (2023). Comparison of antimicrobial resistance among commensal *Escherichia coli* isolated from retail table eggs produced by laying hens from the cage and non-cage housing systems in Western Australia. Antibiotics 12:588. 10.3390/antibiotics12030588 36978454 PMC10044583

[B163] Srednik M. E. Lantz K. Hicks J. A. R M.-S. B. Mackie T. A. Schlater L. K. (2021). Antimicrobial resistance and genomic characterization of salmonella dublin isolates in cattle from the united states. PLoS ONE 16:e0249617. 10.1371/journal.pone.0249617 34547028 PMC8454963

[B164] Surveillance de l'antibiorésistance-Rapport Annuel (2011). Available online at: www.agrireseau.net/documents/8528

[B165] Talavera-González J. M. Talavera-Rojas M. Soriano-Vargas E. Vázquez-Navarrete J. Salgado-Miranda J. (2021). In vitro transduction of antimicrobial resistance genes into *Escherichia coli* isolates from backyard poultry in Mexico. Can. J. Microbiol. 67, 415–425. 10.1139/cjm-2020-0280 33395360

[B166] Tang Y. Fang L. C X. Zhang Q. (2017a). Antibiotic resistance trends and mechanisms in the foodborne pathogen, Campylobacter. Anim. Health Res. Rev. 18, 87–98. 10.1017/S1466252317000135 29166961

[B167] Tang Y. Sahin O. Pavlovic N. LeJeune J. Carlson J. Wu Z. . (2017b). Rising fluoroquinolone resistance in campylobacter isolated from feedlot cattle in the united state. Sci. Rep. 7:494. 10.1038/s41598-017-00584-z 28356558 PMC5428712

[B168] Tate H. Li C. Nyirabahizi E. Tyson G. Zhao S. Rice-Trujillo C. . (2021). A national antimicrobial resistance monitoring system survey of antimicrobial-resistant foodborne bacteria isolated from retail veal in the United States. J. Food Prot. 84, 1749–1759. 10.4315/JFP-21-005 34015113 PMC11586651

[B169] Thakur S. Gray G. C. (2019). The mandate for a global "one health" approach to antimicrobial resistance surveillance. Am. J. Trop. Med. Hyg. 100, 227–228. 10.4269/ajtmh.18-0973 30608047 PMC6367630

[B170] Trends in Veterinary Antibiotic Use in the Netherlands 2005-2011 (2011). Available online at: edepot.wur.nl/214172

[B171] Van Boeckel T. P. Brower C. Gilbert M. Grenfell B. T. Levin S. A. Robinson T. P. . (2015). Global trends in antimicrobial use in food animals. Proc. Natl. Acad. Sci. 112, 5649–5654. 10.1073/pnas.1503141112 25792457 PMC4426470

[B172] Varela-Ortiz D. F. Barboza-Corona J. E. González-Marrero J. León-Galván M. F. Valencia-Posadas M. Lechuga-Arana A. A. . (2018). Antibiotic susceptibility of *Staphylococcus aureus* isolated from subclinical bovine mastitis cases and *in vitro* efficacy of bacteriophage. Vet. Res. Commun. 42, 243–250. 10.1007/s11259-018-9730-4 30043292

[B173] Varga C. Brash M. Slavic D. Boerlin P. Ouckama R. Weis A. . (2018). Evaluating virulence-associated genes and antimicrobial resistance of avian pathogenic *Escherichia coli* isolates from broiler and broiler breeder chickens in Ontario, Canada. Avian Dis. 62, 291–299. 10.1637/11834-032818-Reg.1 30339507

[B174] Varga C. Guerin M. T. Bras M. L. Slavic D. Boerlin P. Susta L. (2019). Antimicrobial resistance in fecal *Escherichia coli* and salmonella enterica isolates: a two-year prospective study of small poultry flocks in Ontario, Canada. BMC Vet. Res. 2019:464. 10.1186/s12917-019-2187-z 31864357 PMC6925488

[B175] Vega-Sánchez V. Barba-León J. González-Aguilar D. Cabrera-Díaz E. Pacheco-Gallardo C. Orozco-García A. (2020). Resistencia antimicrobiana de *Salmonella* spp aisladas de canales de cerdo obtenidas de dos tipos de rastros en Jalisco, México. Rev Mex CiencPecu 11, 1004–1015. 10.22319/rmcp.v11i4.5386

[B176] Velasquez C. Macklin K. S. Kumar S. Bailey M. Ebner P. E. Oliver H. F. . (2018). Prevalence and antimicrobial resistance patterns of salmonella isolated from poultry farms in southeastern united states. Poult. Sci. 97, 2144–2152. 10.3382/ps/pex449 29608757

[B177] Ventola C. L. (2015). The antibiotic resistance crisis: part 1: causes and threats. P T 40, 277–283. 25859123 PMC4378521

[B178] Vounba P. Arsenault J. Bada-Alambédji R. Fairbrother J. (2019). Antimicrobial resistance and potential pathogenicity of *Escherichia coli* isolates from healthy broilers in Québec, Canada. Microb. Drug Resist. 25, 1111–1121. 10.1089/mdr.2018.0403 31038391

[B179] Waldner C. Gow S. Parker S. Campbell J. (2019). Antimicrobial resistance in fecal *Escherichia coli* and *Campylobacter* spp. from beef cows in western Canada and associations with herd attributes and antimicrobial use. Can. J. Vet. Res. 83, 80–89. 31097869 PMC6450164

[B180] Weese J. S. Archambault M. Willey B. M. Hearn P. Kreiswirth B. N. Said-Salim B. . (2005). Methicillin-resistant *Staphylococcus aureus* in horses and horse personnel, 2000-2002. Emerging Infect. Dis. 11, 430–435. 10.3201/eid1103.040481 15757559 PMC3298236

[B181] Williams-Nguyen J. Sallach J. B. Bartelt-Hunt S. Boxall A. B. Durso L. M. McLain J. E. . (2016). Antibiotics and antibiotic resistance in agroecosystems: State of the science. J. Environ. Qual. 45, 394–406. 10.2134/jeq2015.07.0336 27065386

[B182] World Health Organization. (2012). World Health Organization: The Evolving Threat of Antimicrobial Resistance: Options for Action. Available online at: apps.who.int/iris/handle/10665/75389

[B183] Wu R. A. Feng J. Y. M Liu D. Ding T. (2023). Overuse of food-grade disinfectants threatens a global spread of antimicrobial-resistant bacteria. Crit. Rev. Food Sci. Nutr. 64, 6870–6879. 10.1080/10408398.2023.2176814 36756870

[B184] Xu C. Kong L. Gao H. Cheng X. Wang X. (2022). A review of current bacterial resistance to antibiotics in food animals. Front. Microbiol. 13:822689. 10.3389/fmicb.2022.822689 35633728 PMC9133924

[B185] Xu S. Sura S. Zaheer R. Wang G. Smith A. Cook S. . (2016). Dissipation of antimicrobial resistance determinants in composted and stockpiled beef cattle manure. J. Environ. Qual. 45, 528–536. 10.2134/jeq2015.03.0146 27065400

[B186] Yang Y. Ashworth A. J. Willett C. Cook K. Upadhyay A. Owens P. R. . (2019). Review of antibiotic resistance, ecology, dissemination, and mitigation in U.S. broiler poultry systems. Front. Microbiol. 10:2639. 10.3389/fmicb.2019.02639 31803164 PMC6872647

[B187] Yaqoob M. U. Wang G. Wang M. (2022). An updated review on probiotics as an alternative of antibiotics in poultry - a review. Anim. Biosci. 35, 1109–1120. 10.5713/ab.21.0485 35073660 PMC9262730

[B188] Yuan Q. B. Guo M. T. Yang J. (2015). Fate of antibiotic resistant bacteria and genes during wastewater chlorination: implication for antibiotic resistance control. PLoS ONE 10:e0119403. 10.1371/journal.pone.0119403 25738838 PMC4349789

[B189] Zaheer R. Cook S. R. an Barbieri R. Goji N. Cameron A. Petkau A. . (2020). Surveillance of *Enterococcus* spp. reveals distinct species and antimicrobial resistance diversity across a one-health continuum. Sci. Rep. 10:3937. 10.1038/s41598-020-61002-5 32127598 PMC7054549

[B190] Zaheer R. Lakin S. M. Polo R. O. Cook S. R. Larney F. J. Morley P. S. . (2019). Comparative diversity of microbiomes and resistomes in beef feedlots, downstream environments and urban sewage influent. BMC Microbiol. 19:197. 10.1186/s12866-019-1548-x 31455230 PMC6712873

[B191] Zaidi M. B. Dreser A. Figueroa I. M. (2015). A collaborative initiative for the containment of antimicrobial resistance in mexico. Zoonoses Public Health 62, 52–57 10.1111/zph.12166 25418055

